# The Impact of Sequencing and Genotyping Errors on Bayesian Analysis of Genomic Data under the Multispecies Coalescent Model

**DOI:** 10.1093/molbev/msaf184

**Published:** 2025-08-18

**Authors:** Jiayi Ji, Paschalia Kapli, Tomáš Flouri, Ziheng Yang

**Affiliations:** Department of Genetics, Evolution, and Environment, University College London, Gower Street, London WC1E 6BT, UK; Department of Genetics, Evolution, and Environment, University College London, Gower Street, London WC1E 6BT, UK; Natural History Museum, Cromwell Road, London SW7 5BD, UK; Department of Genetics, Evolution, and Environment, University College London, Gower Street, London WC1E 6BT, UK; Department of Genetics, Evolution, and Environment, University College London, Gower Street, London WC1E 6BT, UK

**Keywords:** Bpp, introgression, migration, multispecies coalescent, read depth, species tree

## Abstract

The multispecies coalescent (MSC) model accounts for genealogical fluctuations across the genome and provides a framework for analyzing genomic data from closely related species to estimate species phylogenies and divergence times, infer interspecific gene flow, and delineate species boundaries. As the MSC model assumes correct sequences, sequencing and genotyping errors at low read depths may be a serious concern. Here, we use computer simulation to assess the impact of genotyping errors in phylogenomic data on Bayesian inference of the species tree and population parameters such as species split times, population sizes, and the rate of gene flow. The base-calling error rate is extremely influential. At the low rate of *e* = 0.001 (Phred score of 30), estimation of species trees and population parameters are little affected by genotyping errors even at the low depth of ∼3×. At high error rates (*e* = 0.005 or 0.01) and low depths (less than 10×), genotyping errors can reduce the power of species tree estimation, and introduce biases in estimates of population sizes, species divergence times, and the rate of gene flow. Treating heterozygotes in the sequences as missing data (ambiguities) may reduce the impact of genotyping errors. Our simulation suggests that it is preferable in terms of inference precision and accuracy to sequence a few samples at high depths rather than many samples at low depths.

## Introduction

Advancements in sequencing technologies and in statistical methods of data analysis have greatly expanded access to genome-scale data, enabling a broad research community to apply phylogenomic approaches across a wide range of organisms. As a result, the field of phylogenetics has entered the era of phylogenomics. In genomic sequencing, read depth or simply depth refers to the number of sequencing reads that align to a specific position in the reference genome. While whole-genome sequencing at high depths is ideal, practical constraints, such as sequencing costs, DNA quality, and sample availability, often make it unfeasible to sequence a large number of individuals at high depths. These constraints are particularly acute in studies involving historical museum specimens, rare or endangered taxa, or large comparative studies. As a result, datasets often contain regions of variable read depths, including extensive genomic regions of low depths. In such data, sequencing errors may propagate as genotype-calling errors, despite the application of quality filters designed to remove or mask low-confidence regions (Thawornwattana et al. 2018).

In population genetics, the impact of sequencing errors at different read depths on various analyses such as estimation of demographic parameters, detection of disease variants, etc. has been studied extensively (Nielsen et al. 2011). For example, methods have been developed to correct for biases in estimates of parameters such as the population size parameter θ=4Nμ caused by sequencing errors at low read depths (Fumagalli 2013; Lynch et al. 2014; Salk et al. 2018). However, there does not appear to be any study to examine the effects of sequencing errors on species tree estimation and demographic inference under the multispecies coalescent (MSC) model.

The MSC is a simple extension of the single-population coalescent (Kingman 1982) to multiple species to incorporate the phylogeny (Rannala and Yang 2003). In the last two decades, the model has emerged as the natural framework for analysis of phylogenomic data to address a number of interesting questions in evolutionary biology, such as estimation of species phylogeny in presence of gene tree-species tree conflicts (Liu and Pearl 2007; Heled and Drummond 2010; Yang and Rannala 2014; Edwards et al. 2016; Rannala and Yang 2017), estimation of species divergence times accommodating ancestral polymorphism (Rannala and Yang 2003; Burgess and Yang 2008), inference of interspecific gene flow (Nielsen and Wakeley 2001; Gronau et al. 2011; Lohse et al. 2011; Hey et al. 2018; Wen and Nakhleh 2018; Zhang et al. 2018; Flouri et al. 2020, 2023), and species delimitation (Yang and Rannala 2010; Kornai et al. 2024). Data suitable for analysis under the MSC are short genomic fragments that are far apart. They are referred to as loci but may and may not code for proteins. The fragments are short so that recombination within the locus may be ignored while they are far apart so that the gene genealogies are largely independent (Lohse et al. 2011; Zhu et al. 2022). The model has been formulated assuming no sequencing errors with accurate genotype calls at each locus. At low read depths, base-calling and genotyping errors may potentially lead to biased estimates of species trees, divergence times, and gene flow, but it is unclear what levels of sequencing depths may be a cause for concern.

In this article, we simulate multilocus genomic sequence data under the MSC model including sequencing errors at different read depths to examine the impact of genotyping errors on inference of species trees and estimation of population parameters in the MSC model with gene flow. We develop a Markov-chain model of read depths for sites along a sequence and simulate base-calling and genotype-calling errors in the sequence data. The data with genotyping errors are then analyzed using the Bayesian program Bpp (Yang 2015; Flouri et al. 2018) to infer the species tree and to estimate parameters in the MSC-introgression (MSC-I) or MSC-migration (MSC-M) models, with the genotyping errors ignored, to assess the impact of genotyping errors on MSC-based inference. We also included a few summary methods for inferring the species phylogeny in our comparison.

## Theory: Markov Model of Read Depths to Simulate Genotyping Errors

### Overview

**Fig. 1.**
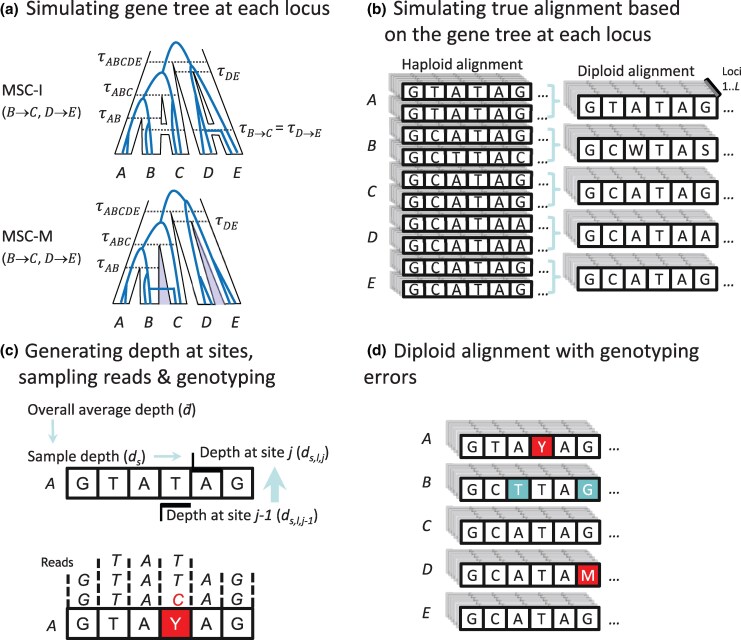
Simulation of multilocus alignments of diploid sequences with genotyping errors by generating read depths at sites and “evolving” sequencing on gene trees using Bpp. a) Simulation of gene trees at multiple loci under the MSC model with or without gene flow. Here, a gene tree with coalescent times for a locus is shown, generated under the MSC-introgression (MSC-I, Flouri et al. 2020) or MSC-migration (MSC-M, Flouri et al. 2023) models (see Fig. 7). b) Simulation of true alignments using the gene trees (with the two haploid sequences for each sample merged into one diploid sequence). c) Simulation of read depths using the beta model described in the article, simulation of reads at sites in the sequence for each locus by binomial sampling of alleles and simulation of genotype calling by ML. d) The resulting alignment of diploid sequences. In c), the diploid sequence from *A* is used as an example, with a base-calling error (T miscalled into C in the reads) and a genotyping error (T miscalled as Y) at the 4th site. In d), genotyping errors in the diploid alignment (either homozygotes miscalled as heterozygotes or heterozygotes miscalled as homozygotes) are shown using shading. Note that in the true diploid sequence for *B* (b), the heterozygotes at two sites, W..S or (A/T)..(G/C), represent the haploid sequences A..G and T..C. Genotyping errors caused the heterozygotes to be miscalled as homozygotes TT and GG (d), and the resulting two haploid sequences, each with T..G at the two sites, are chimeric and differ from the true sequences.

First, we generate aligned correct sequences with no errors at each locus by simulating gene trees under the MSC model and then “evolving” sequences along the gene-tree branches. Then correct sequences are “postprocessed” to introduce genotype-calling errors at the given base-calling error rate (Fig. 1). We develop a Markov model of read depths for sites along a sequence to simulate read depths. Given the read depth and the true genotype at each site, we simulate the reads at the site by multinomial sampling at the given base-calling error rate, and call the genotype at the site by maximum likelihood (ML) (Li 2011). The procedure generates multilocus alignments of unphased diploid sequences which may contain genotyping errors (Fig. 1).

We assume that all samples from the same species have the same average read depth although it is straightforward to allow variable read depths among samples of the same species. Because adjacent sites have a high chance of occurring in the same read, read depths at adjacent sites in the sequence at any locus are expected to be highly correlated. We develop a Markov model to describe the transition of read depths at the adjacent sites in a sequence. We do not simulate sites with zero depth, as they are removed or masked during data processing if the adjacent sites have high depths.

We expect the assumption that read depths along the sequence are Markovian to be unrealistic but it appears to be adequate for our purpose. We do not simulate mapping errors or the use of filters to remove them.

### A Beta-Markov Model of Read Depths Along the Sequence

We use a pair of bounds for read depth: $d_{\min}=2$, $d_{\max}=100$, and use the beta distribution between those bounds to model the read depths for individual sites in a sequence. Let x∼ beta(α,β). This has mean $\frac{\alpha }{\alpha +\beta }$ and variance $\frac{\alpha \beta }{\left(\alpha +\beta {)}^{2}(\alpha +\beta +1\right)}$. Then


(1) $$d=d_{\min}+x\cdot (d_{\max}-d_{\min}),\;d_{\min}\leq d\leq d_{\max},$$

has the 4-parameter beta distribution, with parameters ($\alpha ,\beta ,d_{\min},d_{\max}$). In practice, the read depth is rounded to an integer, as ⌊d+0.5⌋, where ⌊a⌋ is the largest integer that does not exceed *a*. As the bounds $d_{\min}$ and $d_{\max}$ are fixed in our simulation,


(2) $$x=\frac{d-d_{\min}}{d_{\max}-d_{\min}},\;0\leq x\leq 1,$$

and *d* form a one-to-one mapping. We thus treat *x* as a scaled read depth and describe our model using *x* instead of *d* for simplicity.

Let $\bar{d}$ or $\bar{x}$ be the overall average read depth, specified in the simulation. Let ${\bar{d}}_{s}$ or ${\bar{x}}_{s}$ be the average read depth for species/sample *s*, which applies to all loci in all samples from species *s*. This is generated as


(3) $${\bar{x}}_{s}∼\text{beta}(\bar{x}a_{s},(1-\bar{x})a_{s}),$$

where $a_{s}$ is a concentration parameter that describes how much ${\bar{x}}_{s}$ varies among species (with a larger $a_{s}$ representing less variation). Note that ${\bar{x}}_{s}$ has the mean $\bar{x}$ and variance $\bar{x}(1-\bar{x})/(a_{s}+1)$.

Let $x_{slj}$ be the read depth at the *j*th site in the *l*th locus in species/sample *s*. We use a Markov model to simulate the transition of read depths at adjacent sites along the sequence. For the first site (j=1), we have


(4) $$x_{sl1}\;|\;{\bar{x}}_{s}∼\text{beta}({\bar{x}}_{s}a_{p},(1-{\bar{x}}_{s})a_{p}),$$

with mean ${\bar{x}}_{s}$, where $a_{p}$ describes how variable read depths are among positions (sites) in the same sequence (with a larger $a_{p}$ representing less variation). We used $a_{s}=500$ and $a_{p}=1,000$, with more fluctuation between species than between sites.

For site j=2,…, we generate $x_{slj}$ from the beta distribution, $x_{slj}∼$ beta(${\bar{x}}_{slj}a_{p},(1-{\bar{x}}_{slj})a_{p}$), with the mean ${\bar{x}}_{slj}$ specified as a weighted average of the depth at the previous site and the mean depth for the species/sample:


(5) $${\bar{x}}_{slj}=px_{s,l,j-1}+(1-p){\bar{x}}_{s}.$$

Here, parameter *p* determines how strongly correlated read depths are at adjacent sites. We use p=0.9 based on an analysis of sitewise read depths in genomic sequence data (Table 1, model 1). The algorithm generates read depths $d_{sl1},d_{sl2},\ldots$ for sites in the sequence at locus *l* from sample *s*.

**Table 1:** Deviation measuring goodness of fit (*Q*, equation 7) of two models for read depths along the sequence to observed data from two sequenced genomes (Fig. 2)

	Chimpanzee genome (20.26×)		Rabbit genome (4.21×)	
*p*	Model 1	Model 2	Model 1	Model 2
0	0.0023	0.0023	0.0626	0.0626
0.1	0.0022	0.0025	0.0631	0.0695
0.2	0.0021	0.0033	0.0618	0.0779
0.3	0.0019	0.0049	0.0586	0.0879
0.4	0.0017	0.0072	0.0533	0.0995
0.5	0.0014	0.0106	0.0457	0.1128
0.6	0.0011	0.0150	0.0358	0.1277
0.7	0.0008	0.0206	0.0237	0.1444
0.8	0.0005	0.0275	0.0113	0.1628
0.9	0.0002	0.0360	0.0035	0.1830

Note. Model 1 is the beta model based on equation 5, used in our simulation. Model 2 is the alternative model based on the beta distribution described in the text.

We considered an alternative model (Table 1, model 2), in which the read depth at the current site ($x_{slj}$) is assigned the read depth at the previous site ($x_{s,l,j-1}$) with probability *p* and generated from the beta distribution beta(${\bar{x}}_{s}a_{p},(1-{\bar{x}}_{s})a_{p}$) with probability (1−p). This appeared to fit the empirical data less well (see below) and was thus not used. Besides the beta kernel, we also considered a gamma-kernel for Markovian transition of read depths between sites, but it may produce very low read depths (0 or 1), and truncation to apply the bounds ($d_{\min},d_{\max}$) changes the mean, making the model awkward to use.

We processed real genomic data to collect the observed read depths at adjacent sites to assess the fit of our models. Let $f_{xy}$ be the observed frequencies of doublet sites with read depths *x* and *y*, respectively. The probability of observing two adjacent sites with read depths *x* and *y* under our model is


(6) $$e_{xy}=p_{x}p_{xy},$$

where $p_{x}$ is the overall proportion of read depth *x* (or the stationary distribution of the Markov chain), and $p_{xy}$ is the transition probability (i.e. the probability that the read depth for the next site is *y* given that the read depth for the current site is *x*). We can measure the discrepancy between the observed and expected frequencies by


(7) $$Q=E(e_{xy}-f_{xy}{)}^{2}=\sum_{x}\sum_{y}e_{xy}(e_{xy}-f_{xy}{)}^{2}.$$

### Simulating Reads Given Read Depths and True Genotypes

Let ϵ be the base-calling error rate. This important parameter reflects the sequencing technology and may be independently estimated (Meacham et al. 2011; Stoler and Nekrutenko 2021). Currently an error rate of ϵ=0.001, which corresponds to a Phred score of $-10\log_{10}\;\epsilon =30$, is among the best achievable with modern next-generation sequencing technologies, such as HiSeq 2500, HiSeq X Ten, and NovaSeq 6000 (Ma et al. 2019; Stoler and Nekrutenko 2021). An earlier study reported error rates of 0.001–0.01 for Illumina sequencing machines of the time (Lou et al. 2013).

Given ϵ and the read depth $d_{slj}$, we use the true genotype at the position to generate the reads by multinomial sampling. For each read, one of the two alleles at the position is chosen at random and is then read correctly with probability 1−ϵ and incorrectly with probability ϵ. When a read error occurs, one of the three alternative bases is chosen at random (each with probability $\frac{1}{3}$). The base-calling error rate ϵ is assumed to be the same among the reads, independent of the true base. We do not deal with three or four alleles at one position and repeat the simulation for the site if more than two alleles occur. This process produces the reads at the site given the read depth and the true genotype at the site.

#### Calling genotypes given read depths by ML

Given the simulated reads, genotypes were called using ML (Li 2011). Given the data of *k* 1s and (n−k) 0s among the *n* reads, where 0 refers to one allele and 1 the alternative allele, the likelihoods for the three genotypes (GT = 00, 01, and 11) are given by the binomial probabilities as


(8) $$\begin{array}{rl} L(00\;|\;k) & =P(k\;|\;\text{GT}=00)=\left(\begin{array}{c} n\\ k \end{array}\right)(1-\epsilon {)}^{n-k}\epsilon^{k},\\ L(01\;|\;k) & =P(k\;|\;\text{GT}=01)=\left(\begin{array}{c} n\\ k \end{array}\right){\left(\frac{1}{2}\right)}^{n},\\ L(11\;|\;k) & =P(k\;|\;\text{GT}=11)=\left(\begin{array}{c} n\\ k \end{array}\right)(1-\epsilon {)}^{k}\epsilon^{n-k}. \end{array}$$

The genotype achieving the highest likelihood is the inferred (called) genotype.

### Implementation of the Algorithm for Simulating Genotype-calling Errors

The above algorithms for simulating sitewise read depths and for simulating diploid sequences with possible genotyping errors are implemented in Bpp. The option variable seqerr has the following syntax seqerr = 5 0.001 500.0 1000.0 (read depth & base-calling error & a_samples & a_positions), where the four parameters are the average read depth ($\bar{d}$), the base-calling error (ϵ), $a_{s}$, and $a_{p}$, respectively.

The simulation algorithm makes repeated use of binomial sampling to generate reads at individual sites. With the notation of equation 8, given the read depth *n* for a site, the number of the 1 alleles, k=0,1,…,n, has binomial probability P(k|GT), which depends on the read depth *n* and the base-calling error ϵ if the true genotype is a homozygote, and on the read depth *n* only (independently of ϵ) if the true genotype is a heterozygote. In either case *k* has a multinomial distribution with n+1 categories. We set up the alias and look-up tables for different *n* for the alias method for sampling from the multinomial distribution, with the probabilities for categories calculated using equation 8. This method requires generation of one random number to sample a multinomial variable, irrespective of the number of categories (see, e.g. Yang 2014, p. 421).

To simulate a replicate dataset, we sample the average read depth for species *s* (${\bar{d}}_{s}$). We loop through all loci, and for each sequence at each locus generate the read depths for sites. Then, we sample the reads for each site using the alias method and call genotypes by ML. The simulation algorithm seems to be efficient, taking no more time than printing the generated sequence alignments onto the disk.

In our algorithm, the average read depth for species *s* (${\bar{d}}_{s}$) is species-specific, applied to all samples, all sequences, and all sites from that species in the whole dataset. However, ${\bar{d}}_{s}$ may differ among species in the same dataset and among replicate datasets for the same species.

Heterozygotes in diploid sequences are coded using the International Union of Pure and Applied Chemistry (IUPAC) ambiguity codes (e.g. Y stands for a T/C heterozygote). When the data are analyzed by Bpp, the phase variable is used to instruct Bpp to resolve each heterozygote genotype into the two alleles, averaging over all possible resolutions of phase at multiple heterozygous sites in the same sequence using the algorithm of Gronau et al. (2011, see also Huang et al. 2022). In the case of no sequencing or genotyping errors, this algorithm produced results that were nearly equally precise as the use of the fully resolved haploid sequences (Gronau et al. 2011; Huang et al. 2022).

## Results

### Empirical Examination of Read Depths at Adjacent Sites in the Genome

**Fig. 2.**
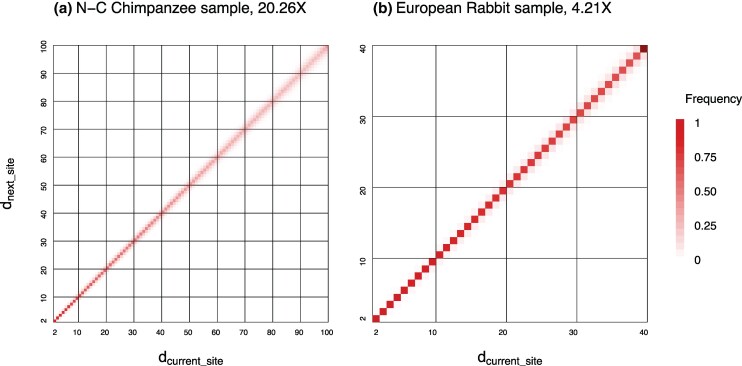
Heat-map representation of empirical transition probabilities of read depths at two adjacent sites (${\hat{p}}_{xy}=f_{xy}/f_{x}$) estimated from a) a Nigeria-Cameroon chimpanzee genome (*Pan troglodytes ellioti*), sequenced on the Illumina HiSeq 2000 platform to an average depth of 20.26× by Prado-Martinez et al. (2013) (NCBI accession: SRX360475) and b) a European rabbit genome (*Oryctolagus cuniculus*), sequenced on the Illumina NovaSeq 6000 platform to an average depth of 4.21× by Andrade et al. (2024) (SRX21096756). The shading represents the observed proportions ($p_{xy}$) of read depth *y* at the next site given the read depth at the current site (*x*), with each row summing to 1.

We used sitewise read depths in sequenced genomes to assess the goodness of fit of our Markov-chain models of read depths along the sequence. The proportions ($f_{xy}$) of site doublets with read depths *x* and *y* for a high-depth chimpanzee genome (average depth 20.26×) and another low-depth rabbit genome (4.21×) were used to generate empirical estimates of transition probabilities (Fig. 2). These suggest strong correlation in read depth between adjacent sites, with high probabilities that the read depth for the next site is identical or very similar to that for the current site. The goodness of fit is measured using the average squared difference, *Q* (equation 7). The results (Table 1) suggest that the conditional-mean model fitted the data better for both datasets. We used this model with the parameter value p=0.9 to simulate read depths along the sequence, given the average read depth. At p=0.9, the model predicts the probability that two adjacent sites in a sequence have the same read depth to be 0.315 and 0.742 for the chimpanzee and rabbit genomes, respectively, compared with the observed values 0.573 and 0.941. Read depths in the real data are more strongly correlated than predicted by the model, indicating that a *P* larger than 0.9, as might be obtained by minimizing *Q* to estimate *P*, might fit these data even better. This is not pursued here. We expect the average read depth to be much more important in our simulation than the correlation (or the non-Markovian nature) of read depths at adjacent sites.

### Species Tree Estimation in Presence of Genotyping Errors

**Fig. 3.**
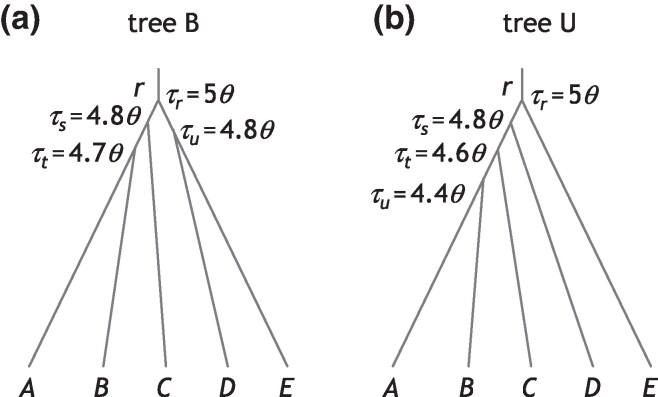
Species trees B and U for five species (A,B,C,D,E) used to simulate data for Bpp estimation of the species tree (the A01 analysis). For a) balanced species tree B, the parameters used in the simulation are $\tau_{r}=5\theta$, $\tau_{s}=4.8\theta$, $\tau_{t}=4.7\theta$, and $\tau_{u}=4.8\theta$. For b) unbalanced species tree U, we used $\tau_{r}=5\theta$, $\tau_{s}=4.8\theta$, $\tau_{t}=4.6\theta$, and $\tau_{u}=4.4\theta$. In each tree, two values of *θ* are used: 0.0025 and 0.01. For analysis using Astral and concatenation/ML, we included an outgroup species (*O*) with a divergence time of 10θ.

**Fig. 4.**
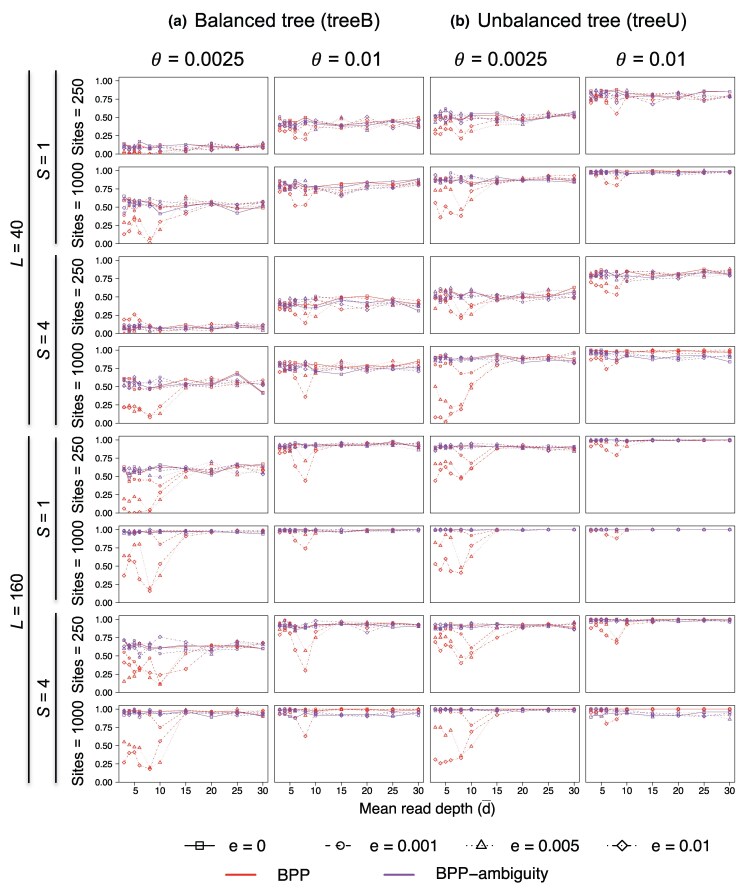
Accuracy of Bpp species tree estimation at different mean read depths ($\bar{d}$) and base-calling error rates (ϵ) for a) the balanced and b) unbalanced species trees, measured by the proportion of replicates in which the inferred species tree by Bpp (the MAP tree) is correct.

We simulated multilocus sequence datasets using the species trees B and U of Fig. 3 to examine the impact of genotyping errors on species tree estimation under the MSC model. Each dataset consists of L=40 or 160 loci, with either S=1 or 4 diploid sequences per species per locus. The Bpp analysis averages over all possible phase resolutions at heterozygote sites in diploid sequences (Gronau et al. 2011; Huang et al. 2022). The maximum *a posteriori* (MAP) species tree is the Bayesian estimate of the true species tree (Rannala and Yang 1996). Accuracy of species tree estimation is measured by the probability that the MAP tree matches the true tree in topology (Fig. 4; see also supplementary fig. S1, Supplementary Material online for the average posterior probabilities for the true tree).

The case of no base-calling errors (ϵ=0) represents the best-case scenario (Fig. 4). We note that genotyping errors may occur at low read depths even in the absence of base-calling errors: when ϵ=0, it is possible for heterozygotes to be miscalled as homozygotes even though it is impossible for homozygotes to be miscalled as heterozygotes. Here, we used the true alignments for ϵ=0 and did not incorporate genotyping errors at ϵ=0.

At the low base-calling error rate of ϵ=0.001, species tree estimation appeared to be little affected by genotyping errors, as accuracy at low depth ($\bar{d}=3,4,5$) was similar to that with no errors (ϵ=0) (Fig. 4). This error rate is of special interest as it is representative of today’s sequencing technology.

At the high base-calling error rates (ϵ=0.01 and 0.005) and low read depths, species tree estimation was affected by genotyping errors, with the accuracy at $\bar{d}=3$–8 being considerably lower compared with accuracy achieved using data of no errors (ϵ=0). When the average read depth reached 15, the impact of genotyping errors became unimportant even at the high ϵ.

**Fig. 5.**
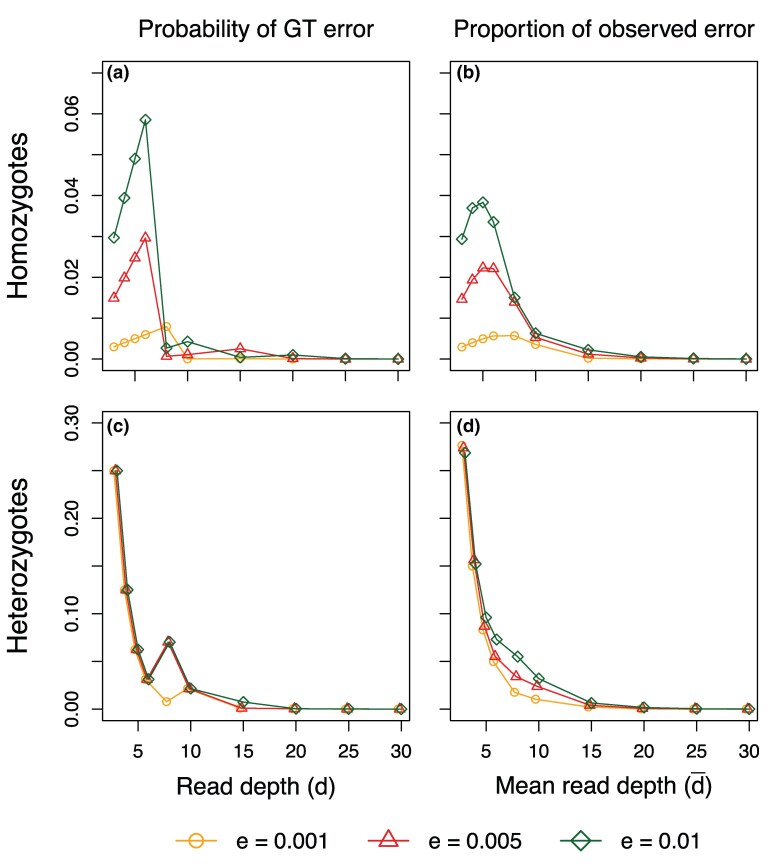
a, c) Probability of genotyping error given the genotype (homozygote or heterozygote), the base-calling error rate (ϵ), and the read depth (*d*) (Thawornwattana et al. 2022, equation 2), and b, d) the observed error rate in simulations at the average read depth. In our simulation, the read depth at each site in the sequence varies around the average read depth according to the beta-Markov model. Note that when ϵ=0 the genotyping error rate is 0 for homozygotes and $(\frac{1}{2}{)}^{d-1}$ for heterozygotes.

Note that at the high base-calling error rate (ϵ=0.01), accuracy of species tree estimation at read depth 8 was often lower than at read depths 3–5 (Fig. 4). This result may be counter-intuitive and is due to the fact that because of the discrete nature of read depth *d*, genotyping error is not a monotonically decreasing function of *d* and may increase when *d* increases: for example, at ϵ=0.01, genotyping errors for heterozygotes are higher at d=8 than at d=5 (Fig. 5; see also Thawornwattana et al. 2022, Fig. 1). Also at high base-calling errors (ϵ=0.01 and 0.005), accuracy can be lower when S=4 samples were included in the data than when only one sample (S=1) was used (e.g. tree U with θ=0.0025, with either L=40 or 160 loci with the sequence length N=1,000).

Nevertheless, for most of our simulation settings involving genotyping errors (with ϵ>0), increasing the amount of data (the number of loci *L*, the number of samples per locus *S*, the sequence length *N*, and the mutation rate *θ*) improved the accuracy of species tree estimation by Bpp (Fig. 4). In particular, accuracy improved when the number of loci increased from L=40 loci to 160, suggesting that accuracy may improve further if L→∞. To see whether species tree estimation by Bpp is statistically consistent despite genotyping errors, we simulated datasets with increasing numbers of loci (*L*), using species tree B at the low mutation rate (θ=0.0025), a setting in which the observed accuracy was among the lowest (Fig. 4). The results are shown in supplementary fig. S2, Supplementary Material online. In general, accuracy improved with increasing data size *L*, although the trend is not clear-cut at the depth $\bar{d}=10$. While it may be possible to find very hard species trees for which the method is inconsistent at low read depths and high genotyping errors, the main effect of genotyping errors appears to be a dilution of phylogenetic information so that a much greater amount of data is needed to estimate the species tree with high confidence in presence of genotyping errors.

Our simulation experiment is designed to simplify the assessment of the effects of different factors: the number of loci (*L*), the number of samples per locus (*S*), the sequence length (*N*), and the mutation rate (*θ*). A previous simulation assuming no sequencing errors or model violations has found that for species tree estimation, the most important factor is the number of loci (*L*), followed by the sequence length (*N*) and the mutation rate (*θ*), while the number of sequences (*S*) is the least important factor (Huang et al. 2020, Table 6). Here, the results are consistent with the previous study. We note that in the smallest datasets (with L=40 loci, S=1 diploid sequence per species, and N=250 sites), accuracy was low, indicating a lack of information (Fig. 4). Note that in our simulation the species split times (*τ*) are proportional to the population size *θ*, so that changing *θ* simply scales the branch length on the species tree without changing the shape of the tree, and different *θ* values mimic the use of genomic regions with different mutation rates (e.g. noncoding DNA vs. exons).

Also accuracy was higher for the unbalanced tree U (Fig. 3) than for the balanced tree B. This was due to our choice of the internal branch lengths: in tree B, the three internal branch lengths have the lengths 0.1θ, 0.2θ, and 0.2θ, whereas in tree U, all three internal branches have the length 0.2θ (Huang et al. 2020).

Next, we consider the Bpp analysis treating called heterozygotes as ambiguities or missing data (Fig. 4, Bpp-ambiguity). The approach reduced the impact of genotyping errors at the high error rates (ϵ=0.01,0.005) and low read depths ($\bar{d}=3,4,5,6,8,10$) considerably. With this approach, the results were very similar at different base-calling error rates and at different average read depths, and similar to the best-scenario results at ϵ=0.

**Fig. 6.**
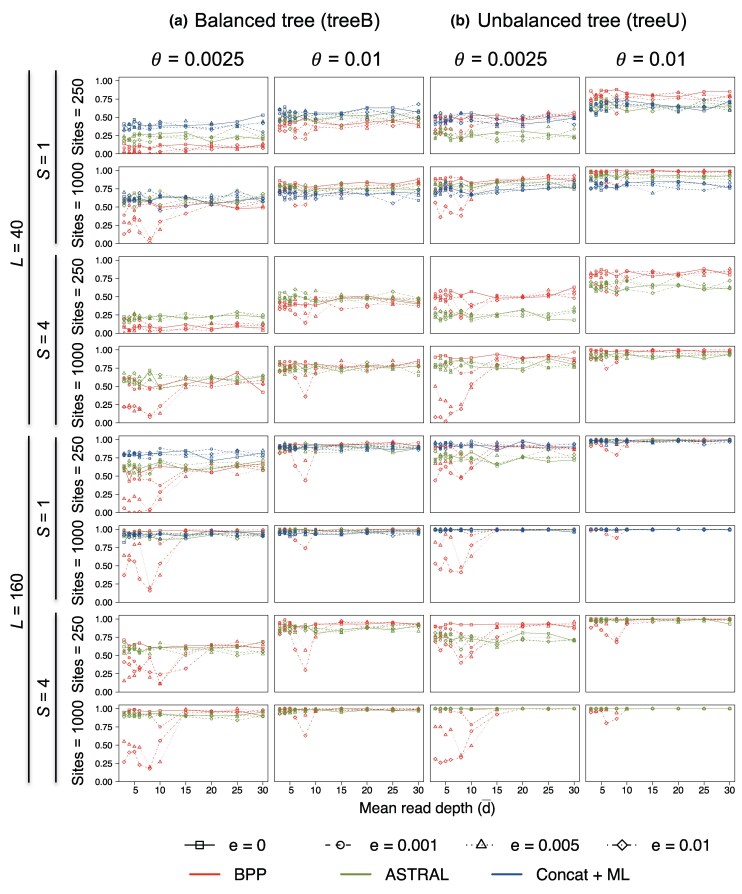
Accuracy of species tree estimation using Bpp, Astral, and concatenation/ML for a) balanced and b) unbalanced species trees for five species. The results are shown separately for the three methods in Fig. 4 and supplementary fig. S3, Supplementary Material online. Concatenation/ML is applied to the case of one (diploid) sequence per species (S=1) only.

The simulated data were also analyzed using Astral and concatenation/ML to estimate the species tree. As these methods do not infer the root of the species tree, we included a sequence from a distant outgroup species (*O*) to root the tree, for comparison with Bpp (Fig. 3). For Astral analysis, we used Raxml to reconstruct the gene tree for each locus under the JC model and then used Astral to generate the species tree. Raxml treats the diploid sequence with heterozygotes as a haploid sequence with ambiguities, using the same approach as Bpp-ambiguity. The results are summarized in Fig. 6 (see also supplementary fig. S3, Supplementary Material online). Overall, Astral and concatenation/ML appeared to be robust to genotyping errors in the simulation settings used here. Performance was nearly identical at different read depths and at different base-calling error rates. The two methods performed better than Bpp and were similar to Bpp-ambiguity, suggesting that treating heterozygotes as ambiguities had the effect of ameliorating the impact of sequencing errors at low depths.

### Parameter Estimation Under the MSC-I Model

**Fig. 7.**
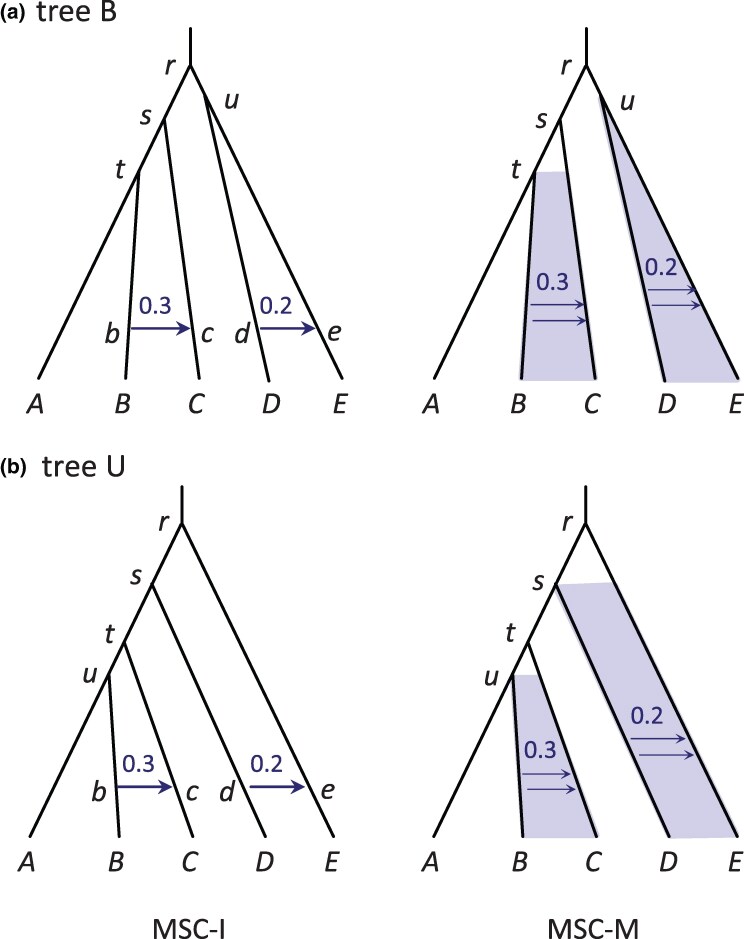
a) Balanced species tree B and b) unbalanced species tree U for five species with gene flow used in the simulation to evaluate Bayesian parameter estimation. Gene flow is modeled using either MSC-I or MSC-M. The parameters for tree B are $\tau_{r}=5\theta$, $\tau_{s}=4\theta$, $\tau_{t}=3\theta$, $\tau_{u}=4.5\theta$, $\tau_{b}=\tau_{c}=\theta$, and $\tau_{d}=\tau_{e}=\theta$, while those for tree U are $\tau_{r}=5\theta$, $\tau_{s}=4\theta$, $\tau_{t}=3\theta$, $\tau_{u}=2.5\theta$, $\tau_{b}=\tau_{c}=\theta$, and $\tau_{d}=\tau_{e}=\theta$. We used two values for *θ*: 0.0025 or 0.01. In the MSC-I model, we used $\varphi_{bc}=0.3$ and $\varphi_{de}=0.2$, while in the MSC-M model, we used $M_{bc}=m_{bc}N_{C}=0.3$ and $M_{de}=0.2$.

We simulated data using the balanced and unbalanced species trees (B and U) for five species with two gene-flow events of Fig. 7 and analyzed the data using Bpp to estimate parameters in the model. In this subsection, we discuss the results under the MSC-I model. The true introgression probabilities were $\varphi_{bc}=0.3,\varphi_{de}=0.2$.

**Fig. 8.**
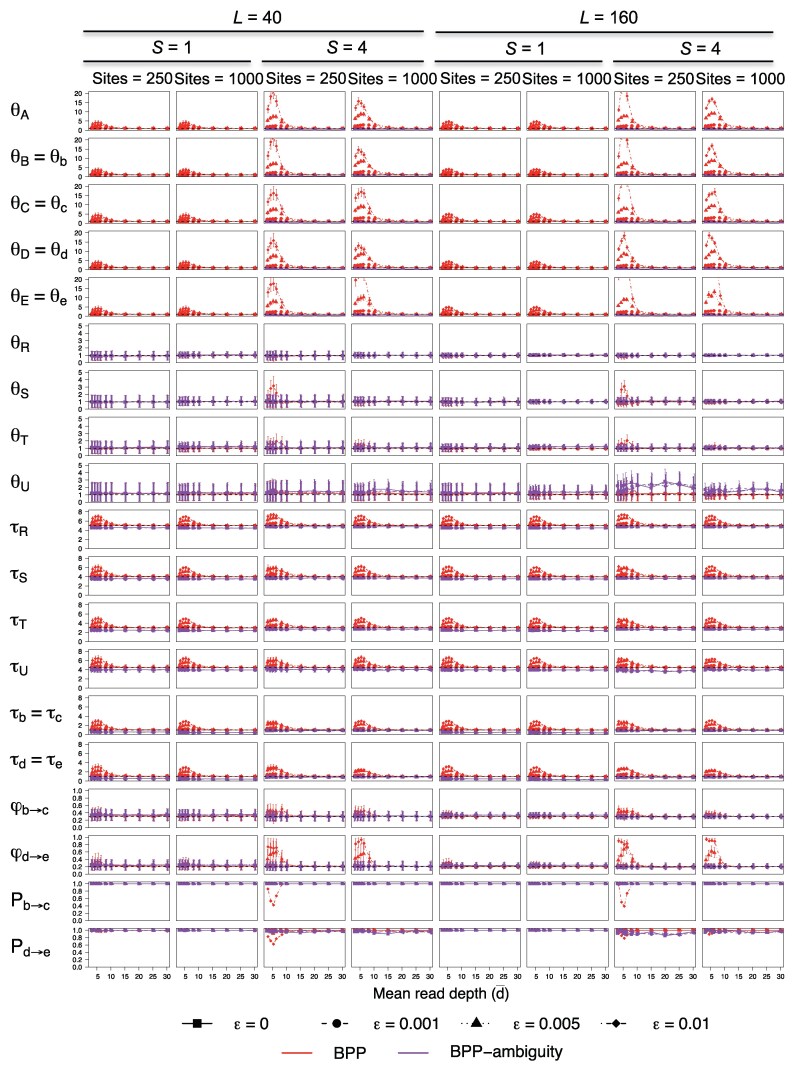
Average posterior means and 95% HPD CIs for parameters in data simulated and analyzed under the MSC-I model of tree B (Fig. 7a) with θ=0.01. Dashed lines indicate true parameter values (*τ* and *θ* are multiplied by 100). $P_{b\to c}$ is the power of the Bayesian test or the proportion of replicate datasets in which the Bayesian test inferred b→c introgression at the 1% level (with $B_{10}\geq 100$).

The average posterior means and highest probability density (HPD) credibility intervals (CIs) are presented in Fig. 8 and supplementary figs. S4–S6, Supplementary Material online. We also applied the Bayesian test of gene flow, calculating the Bayes factor $B_{10}$ via the Savage–Dickey density ratio (Ji et al. 2023), with the test considered significant if $B_{10}>100$. The power of the test, or the proportion of replicate datasets in which gene flow is inferred, is summarized in Fig. 8 and supplementary figs. S4–S6, Supplementary Material online as $P_{b\to c}$ and $P_{d\to e}$.

We first discuss the estimation of the rates of gene flow and the Bayesian test of gene flow, before estimation of other parameters in the model.

At the low base-calling error rate of ϵ=0.001, estimates of $\varphi_{bc}$ and $\varphi_{de}$, even at low depth ($\bar{d}=3,4,5$), were similar to those obtained in the case of no genotyping errors (ϵ=0) (Fig. 8 and supplementary figs. S4–S6, Supplementary Material online). Biases were slightly larger at the lower mutation rate (θ=0.0025) than at the high rate (θ=0.01) but overall the impact of genotyping errors was small even at low depths.

At high base-calling error rates (ϵ=0.01 and 0.005) and low depths (with $\bar{d}<10$), estimates of introgression probabilities ($\varphi_{bc}$ and $\varphi_{de}$) involved large uncertainties and biases (Fig. 8 and supplementary figs. S4–S6, Supplementary Material online). Biases were far more pronounced when S=4 samples per species were included in the data than when S=1, and at the lower mutation rate than at the high mutation rate (θ=0.0025 vs. 0.01). While genotyping errors most often caused positive biases in $\varphi_{bc}$ and $\varphi_{de}$, negative biases were observed in some cases (e.g. supplementary fig. S4, Supplementary Material online, $\varphi_{bc}$, S=4). The power of the Bayesian test also suffered in such cases, consistent with the wide CIs for the introgression probability. The bias largely disappeared when the average read depth reached $\bar{d}=10$. Power of the test was in general high, except for the d→e introgression in tree B, in small datasets of L=40 loci, with S=1 or 4 samples per species and at the lower mutation rate θ=0.0025. Note that in tree B, the d→e gene flow is between sister lineages whereas the other gene-flow events are between nonsister lineages: in general gene flow between sister lineages is harder to detect than between nonsister lineages.

Should one sequence a few samples at high depths or many samples at low depths? For example, the two scenarios S=1 with d=20 and S=4 with d=5 may involve comparable sequencing effort or cost. The answer to this question is clear-cut: a few high-depth samples are preferable over many low-depth samples. Indeed at d=5, use of S=4 samples exacerbated the bias and was worse than having one sample (S=1 at d=5), which was in turn worse than one sample sequenced at high depths (S=1 at d=20).

Estimation of other parameters such as population sizes and species split times were also affected by genotyping errors at high base-calling error rates (ϵ=0.01 and 0.005) and low read depths ($\bar{d}<10$) (Fig. 8 and supplementary figs. S4–S6, Supplementary Material online). Population sizes for modern species ($\theta_{A},\theta_{B},\theta_{C},\theta_{D},\theta_{E}$) had wide 95% CIs and large positive biases, while those for ancestral species were much better estimated. Speciation/introgression times ($\tau_{R},\tau_{S},\tau_{T},\tau_{U},\tau_{b},\tau_{d}$ under MSC-I) were also overestimated. Both the uncertainties and the positive biases in *θ*s and *τ*s were more pronounced at the lower mutation rate than at the high rate (θ=0.01 vs. 0.0025). Again biases were reduced to unimportant levels when the average read depth reached $\bar{d}=10$.

Treating heterozygotes as missing data (Bpp-ambiguity) reduced the bias in parameter estimation caused by genotyping errors at low depths. With heterozygotes treated as ambiguities, there is little difference between S=1 and S=4. At high read depths ($\bar{d}\geq 10$), the standard Bpp analysis of diploid sequences was little affected by genotyping errors. In contrast, Bpp-ambiguity systematically underestimated population sizes and species divergence times (*θ*s and *τ*s), with ancestral population sizes having extremely wide intervals (Fig. 8, supplementary figs. S4–S6, Supplementary Material online). However at high base-calling error rates and low depth, the Bpp-ambiguity approach tended to ameliorate the impact of genotyping errors.

### Parameter Estimation Under the MSC-M Model

**Fig. 9.**
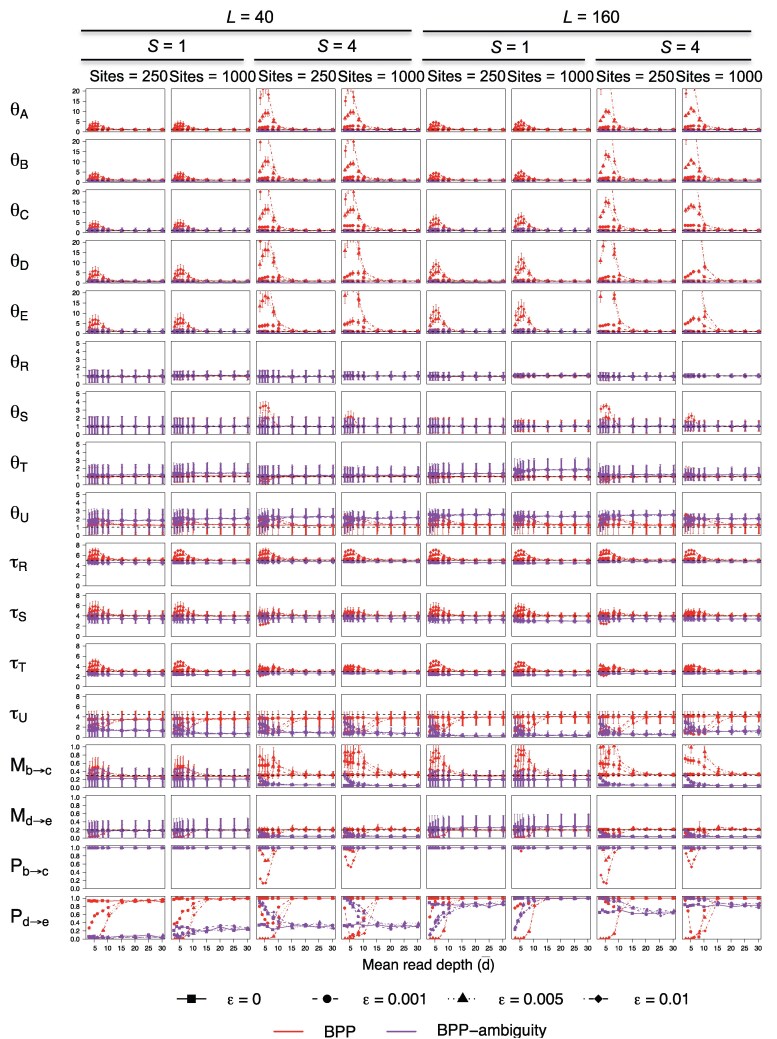
Average posterior means and 95% HPDs for parameters under the MSC-M model of tree B (Fig. 7a) with θ=0.01. $P_{b\to c}$ and $P_{d\to e}$ is the power of the Bayesian test of gene flow (at the cutoff $B_{10}\geq 100$). See legend to Fig. 8.

We used the species trees B and U with continuous migration of Fig. 7 to simulate data to examine the impact of genotyping errors on estimation of parameters under the MSC-M model. The true population migration rates were $M_{bc}=0.3,M_{de}=0.2$. Here, the population migration rate $M_{XY}=m_{XY}N_{Y}$ is the expected number of X→Y migrants per generation, where $m_{XY}$ is the proportion in the recipient population *Y* of immigrants from *X*, and where $N_{Y}$ is the population size of *Y*. The average posterior means and HPD CIs for parameters in the model are shown in Fig. 9, and supplementary figs. S7–S9, Supplementary Material online. The Bayesian test of migration was applied by calculating the Bayes factor $B_{10}$ via the Savage–Dickey density ratio (Ji et al. 2023), with the null region defined as M<0.005. The power of the test is presented in Fig. 9 and supplementary figs. S7–S9, Supplementary Material online as $P_{b\to c}$ and $P_{d\to e}$.

We first discuss the estimation of the rate of gene flow (*M*) and the Bayesian test of migration. At the lower base-calling error rate (ϵ=0.001), biases in migration rates ($M_{bc}$ and $M_{de}$) were small. The results were very similar to those obtained with no genotyping errors in settings of high mutation rate (θ=0.01) and one sample per species (S=1). Biases in parameter estimates were slightly larger for the low mutation rate (θ=0.0025) with S=4 samples per species.

At high base-calling error rates (ϵ=0.01 and 0.005) and low read depths (with $\bar{d}<15$), estimates of $M_{bc}$ and $M_{de}$ involved large uncertainties and biases (Fig. 9 and supplementary figs. S7–S9, Supplementary Material online). The biases were more pronounced for S=4 samples per species than for S=1. On the balanced tree B, the migration rate $M_{bc}$ between nonsister lineages had positive biases while $M_{de}$ for migration involving sister lineages had negative biases. The difference is somewhat surprising. The impact on the power of the Bayesian test was consistent with the effect on estimation of the migration rate: genotyping errors caused underestimation of $M_{de}$ and reduced power in the Bayesian test for detecting the d→e migration. On the unbalanced tree U, both $M_{bc}$ and $M_{de}$ are for migration between nonsister sisters, and both rates were overestimated with elevated power for the Bayesian test. Genotyping errors at high base-calling error rates (ϵ=0.01 and 0.005) and low depths appeared to affect the MSC-M model more than the MSC-I model. Biases became nonsignificant when the average read depth reached 15×.

As found in the MSC-I model, parameter estimation and Bayesian test of gene flow were much better at S=1 with d=20 than at S=4 with d=5: a few high-depth samples are far better than many low-depth samples for inference under the MSC-M model.

The impacts of genotyping errors on estimation of other parameters such as population sizes and species split times under the MSC-M model (Fig. 9 and supplementary figs. S7–S9, Supplementary Material online) were similar to that under the MSC-I models discussed above. Genotyping errors at high base-calling error rates (ϵ=0.01 and 0.005) and low depths caused extremely large positive biases in population sizes for modern species ($\theta_{A},\theta_{B},\theta_{C},\theta_{D},\theta_{E}$). Species split times had positive biases as well but the biases were much smaller. The positive biases in *θ*s and *τ*s were more pronounced at the lower mutation rate than at the high rate (θ=0.01 vs. 0.0025), measured on a relative scale (e.g. by bias over mean ratio). Ancestral population sizes ($\theta_{R},\theta_{S},\theta_{T},\theta_{U}$) had little bias.

At the high base-calling error rates (ϵ=0.01 and 0.005), biases in species split times (*τ*) became unimportant when the average read depth reached $\bar{d}=10$, while for modern *θ*s, biases persisted until $\bar{d}=15$.

Treating heterozygotes as missing data (ambiguities) reduced the bias in estimates of the migration rates ($M_{bc},M_{de}$) at low depth for data of one sample per species (S=1). However, the estimates had large uncertainties, and the power of the Bayesian test might be low. For data of S=4 samples per species, this approach caused serious underestimates of $M_{bc}$ and $M_{de}$, and also reduced the power of the Bayesian test to nearly 0. With this approach, the different read depths showed nearly identical results.

Overall, genotyping errors at low depths appeared to have affected the analysis under the MSC-M model more than the analysis under the MSC-I model. Also under the MSC-M model, the approach of treating heterozygotes as ambiguities appeared to be less effective than under MSC-I. If the sequencing error rate is low (e.g. ϵ=0.001), it appears advisable to ignore genotyping errors since their impact was not large.

### The Effects of Two Types of Genotyping Errors

We note that Bpp analysis of sequence data with genotyping errors constitutes Bayesian inference under misspecified models. While it is not surprising that model misspecification may cause systematic biases in parameter estimation and loss of power of the Bayesian test, it is often hard to understand the direction and magnitude of such biases. Here, we ask whether systematic errors in parameter estimation seen in our simulations (Figs. 8, 9, and supplementary figs. S4–S9, Supplementary Material online) are mostly caused by (a) homozygotes miscalled as heterozygotes (hom-err) or (b) heterozygotes miscalled as homozygotes (het-err). To answer this question, we modified our simulation algorithm to generate data with only one type of genotyping errors present. Together with the data of true genotypes and data containing both types of errors, we have four settings, referred to as “no-err,” “hom-err,” “het-err,” and “both-err.” For each setting, we simulated 3×2=6 new datasets, for ϵ=0.001,0.005,0.01 and with either hom-err or het-err. We used species tree B, high mutation rate (θ=0.01), L=160 loci, S=4 samples per species, N=1,000 sites, and read depth d=5; under these settings Bpp produced highly biased parameter estimates at the high error rate when both types of errors were present (Figs. 8 and 9). As the datasets were large and informative with little variation among replicates, we simulated only one replicate for each setting. Each dataset was analyzed using Bpp and Bpp-ambiguity, as before.

The results are summarized in supplementary fig. S10, Supplementary Material online. Parameter estimation showed very similar performance between hom-err and both-err, and between het-err and no-err, suggesting that the errors of homozygotes being miscalled as heterozygotes (hom-err) are responsible for biases in parameter estimation under the MSC-I and MSC-M models seen in our study. The proportions for hom-err and het-err errors in the data are expected to be $(1-\theta )\times e_{\mathrm{hom}}$ and $\theta \times e_{\mathrm{het}}$, respectively, where $e_{\mathrm{hom}}$ and $e_{\mathrm{het}}$ are genotyping error rates for homozygotes and heterozygotes, respectively (Fig. 5a and c). At the read depth d=5 and the base-calling error rate ϵ=0.01, these proportions are 0.04852 and 0.00063, with hom-err being 77 times more common than het-err. The proportions observed in the simulations at the average read depth of $\bar{d}=5$ are 0.03796 and 0.00095, with a 40× difference. Note that in the simulation read depths vary among sites according to the beta-Markov model.

The two types of errors may have different effects on inference under the MSC models. When a heterozygote is miscalled as a homozygote (het-err), there may be two effects that tend to cancel out. First, a het-err error should cause underestimation of heterozygosity or divergence. Second, if two heterozygote sites in the same sequence are both miscalled as homozygotes, the heterozygote phase may be erroneously resolved, generating chimeric haploid sequences which do not exist in the real world and which look different from other sequences at the locus, thus inflating sequence variation at the locus (Fig. 1). This is a similar effect to the phasing errors when a diploid sequence with multiple heterozygote sites is resolved into haploid sequences, for which the approach of treating heterozygotes as ambiguities (Bpp-ambiguity) was also found to ameliorate the systematic errors (Andermann et al. 2019). The two het-err effects are in opposite directions, which may explain the high similarity between het-err and no-err (supplementary fig. S10, Supplementary Material online).

When a homozygote is miscalled as a heterozygote (hom-err), a truly constant site may become a variable site, and the erroneous nucleotide has to be accounted for in the model, inflating both within-species polymorphism and between-species divergence. We suggest that this effect may be responsible for the serious overestimation of population sizes and species divergence times (*θ*s and *τ*s) seen in our simulation (Figs. 8 and 9 and supplementary figs. S4–S9, Supplementary Material online). The same effect may also explain the larger biases observed when S=4 individuals were sampled per species than when only S=1 individual was sampled, and why biases are larger at the lower mutation rate (θ=0.0025 vs. θ=0.01). Note that in our simulation genotyping error rates are independent of the species tree (B vs. U), the mutation rate, and the model of gene flow. At a lower mutation rate, a larger proportion of variants in the data will be errors instead of true mutations; in other words at higher sequence similarity variants caused by genotyping errors will look more surprising and will more seriously inflate the apparent polymorphism and species divergence leading to more serious overestimation of *θ*s and *τ*s.

The approach of treating heterozygotes as ambiguities (Bpp-ambiguity) is found useful to ameliorate the systematic errors, because erroneous variants caused by homozygotes miscalled as heterozygotes (hom-err) as well as chimeric sequences caused by random heterozygote resolution (het-err) are treated as missing data.

## Discussion

### Impacts of Genotyping Errors in Phylogenomic Analyses

In this article, we have developed a model for simulating read depths for sites in a sequence and used it to assess the impact of base-calling errors and genotyping errors on demographic inference under the MSC model. Overall we find that species tree estimation under the MSC is quite robust to genotyping errors at low read depths and high sequencing errors. If the base-calling error rate is low at ϵ=0.001 as is typical with today’s technology, the impact of genotyping errors on species tree estimation appears to be minimal even at the low depths of 3× or 5× (Fig. 4). When data quality is a concern, it may be advisable to use the approach of treating heterozygotes as missing data to see whether it produces different results from the standard analysis of unphased diploid sequences (which averages over phase resolutions at multiple heterozygote sites in each diploid sequence).

We find that estimation of population parameters under the MSC model is more sensitive than species tree estimation to genotyping errors at high base-calling errors and low read depths. At high base-calling errors (ϵ=0.005 or 0.01), a minimum of 10× or 15× depth may be needed to reduce the impact of genotyping errors. Genotyping errors at high base-calling errors and low read depths cause serious overestimation of population sizes for extant species and overestimation of species divergence times (*θ*s and *τ*s), although the effects on the rate of gene flow (φ under MSC-I and *M* under MSC-M) are more complex. Our analysis suggests that biases in parameter estimation are mostly caused by genotyping errors of homozygotes being miscalled as heterozygotes rather than by errors of heterozygotes miscalled as homozygotes.

The approach of treating heterozygotes as missing data (Bpp-ambiguity) may ameliorate the impact of genotyping errors, especially for analysis under the MSC-I model. We find that sequencing a few samples at high depths provides better inference precision and accuracy than sequencing many samples at low depths.

It may be useful to develop multisample genotype-calling methods under the multispecies coalescent model to improve genotyping quality at low read depths. If the species are closely related, even multisample genotype-calling procedures developed for population data (from one species) may improve genotyping quality. More research in this area is needed. Recently, Zhang and Nielsen (2025) developed a method called Waster for inferring species trees using low-depth genomic data, based on the Caster method of Zhang et al. (2025). These are summary methods that use genome-wide site-pattern counts and ignore information in the variation of genealogical histories across the genome. They can estimate the species tree topology but not population demographic parameters such as population sizes and species split times. In the likelihood framework, Gronau et al. (2011) developed the BSNP method, which infers genotypes at each site by using information in the aligned bases, base-call quality scores, and mapping quality scores produced by BWA (Li and Durbin 2009). The algorithm appears to be tailored to human genomic sequence data and does not appear to be adapted to genomic data from other species. If low-depth sequence data are common, it may be worthwhile to implement probabilistic models to accommodate sequencing and genotyping errors in genome sequences at low depths.

### Limitations of Our Simulation and Impact of Genotyping Errors

Here, we discuss a few limitations of our simulation model and of our simulation. First read depths are assumed to be Markovian along the sequence; that is, the read depth at next site depends on the read depth at the current site, but not on read depths at previous sites. This assumption may be highly unrealistic. It should be simple to incorporate high-order dependence in the model. However the objective of our study is to assess the impact of genotyping errors on inference under the MSC. For that purpose, the average read depth should be much more important than the correlation and Markov dependence of read depths at adjacent sites, in particular as read depths at adjacent sites are highly correlated.

Our simulation model does not account for mapping or alignment errors, which may be a major source of sequencing errors in low-depth genomic data. In particular, if genomes are sequenced from different species and if the reference genome is from a distantly related species, mapping errors may be very important. Also we assumed that genotype calling is based on reads for each sample. Use of multiple samples from the same species to call genotypes is known to reduce genotyping errors. Methods for calling genotypes using multiple-sample reads from several species are yet to be developed.

Despite limitations such as these, we suggest that our simulation provides useful guidelines for phylogenomic studies to infer histories of species divergence and gene flow accounting for the coalescent process, when the sequencing depth is low.

## Materials and Methods

### Simulation to Examine Species Tree Estimation

To examine the impact of genotyping errors on estimation of the species tree topology, we simulated multilocus sequence data under the MSC model with no gene flow assuming species trees B or U of Fig. 3, from Huang et al. (2020, see also Zhu et al. 2022). For tree B, the parameters were $\tau_{r}=5\theta$, $\tau_{s}=4.8\theta$, $\tau_{t}=4.7\theta$, and $\tau_{u}=4.8\theta$. For tree U, they were $\tau_{r}=5\theta$, $\tau_{s}=4.8\theta$, $\tau_{t}=4.6\theta$, and $\tau_{u}=4.4\theta$. Two values were used for *θ*: 0.0025 and 0.01. In our setup, species split times (*τ*) are proportional to *θ*, so that different *θ*s represent different mutation rates or the use of different types of genomic data with different mutation rates (e.g. noncoding DNA vs. exons).

Each replicate dataset consisted of L=40 or 160 loci, with either S=1 or 4 diploid sequences per species per locus, and either N=250 or 1,000 sites in the sequence. The number of replicate datasets was 100. Alignments of correct haploid sequences were generated by simulating gene trees with branch lengths (coalescent times) under the MSC model (Rannala and Yang 2003) and then “evolving” sequences along the branches of the gene tree according to the JC model (Jukes and Cantor 1969). Sequences at the tips of the gene tree constituted data of correct sequences with no genotyping errors at the locus (Fig. 1). We then introduced genotyping errors by simulating read depths at sites in each sequence using the beta-Markov model and then simulating reads at each site by using the read depth and the base-calling error rate through multinomial sampling. The reads at each site were then used to call the genotype using ML. We used four values for the base-calling error rate: ϵ=0 (no error), 0.001, 0.005, 0.01, and 10 values for the average read depth $\bar{d}=3$, 4, 5, 6, 8, 10, 15, 20, 25, 30. Data of both true diploid sequences and sequences with genotyping errors were generated using the simulate option of Bpp (Yang 2015; Flouri et al. 2018). Heterozygotes were coded using IUPAC ambiguity codes.

Each dataset of unphased diploid sequences, with possible genotyping errors, was analyzed using Bpp to estimate the species tree. The likelihood calculation averages over all possible phase resolutions of multiple heterozygote sites in each sequence, using the algorithm of Gronau et al. (2011). The subtree-pruning-and-regrafting (SPR) algorithm was used to move between species trees (Rannala and Yang 2017; Flouri et al. 2018). We integrated out *θ*s analytically through the use of the conjugate inverse-gamma priors (Flouri et al. 2018), which may help with Markov chain Monte Carlo (MCMC) mixing. We assigned inverse-gamma (IG) priors on the age of the root ($\tau_{0}=\tau_{r}$) and the population size parameters (*θ*) in the MSC model: if the true *θ* value is 0.01, θ∼ IG(3, 0.02) and $\tau_{0}∼$ IG(3, 0.1); and if the true *θ* value is 0.0025, θ∼ IG(3, 0.005) and $\tau_{0}∼$ IG(3, 0.025), with the mean matching the truth. As the starting species tree affects the time taken to reach stationarity, but not the mixing efficiency of the Markov chain after the burn-in, we used the true species tree as the starting tree. We calculated the posterior probabilities for the species tree and clades to measure performance.

We also tested the approach of treating heterozygotes in a diploid sequence as an undetermined nucleotide in a haploid sequence (e.g. with the heterozygote Y meaning both T and C treated as an unknown nucleotide that is either T or C) (Andermann et al. 2019; Huang et al. 2022). This is referred to as Bpp-ambiguity. When there are no genotyping errors, this approach makes use of about half of the data of diploid sequences, and may also introduce biases (such as underestimation of heterozygosity). However, Andermann et al. (2019) found that this approach ameliorated the impact of incorrect heterozygote phase resolutions in so-called “haploid consensus sequences” generated in genome-sequencing projects (which in effect resolve the phase of multiple heterozygotes in one diploid sequence at random) (Huang et al. 2022). Here, we applied the approach to examine whether it might reduce the impact of genotyping errors.

The simulated data were also analyzed using Astral and concatenation/ML to estimate the species tree. The sequence data included an outgroup species (*O*) to root the tree, which diverged from the ingroup species at time τ=10θ (Fig. 3). For Astral analysis, we used Raxml to reconstruct the gene tree for each locus under the JC model (Jukes and Cantor 1969) and then used Astral to generate the species tree. There is no algorithm in Raxml to deal with unphased diploid sequences properly; instead it treats the diploid sequence with heterozygotes as a haploid sequence with ambiguities (exactly as does Bpp-ambiguity). The concatenation method was applied to the case of S=1 diploid sequence per species only. Sequences from all loci were concatenated and the super-alignment was analyzed using Raxml to generate one tree, which was the estimate of the species tree. Again heterozygotes were treated as ambiguities.

### Simulation for Estimating Divergence Times, Population Sizes, and Rates of Gene Flow

We used species trees B and U of Fig. 7, each with two gene-flow events, to simulate data to examine the impact of genotyping errors on estimation of parameters in the MSC model, such as the species divergence times (*τ*), population sizes, and the rates of gene flow (the introgression probability or the migration rate), with the species tree fixed. For each species tree, we used two models of gene flow: the discrete MSC-I model which assumes that introgression/hybridization occurs at a time point in the past (Flouri et al. 2020), and the continuous migration (MSC-M) model which assumes that gene flow occurs over an extended time period (Flouri et al. 2023). The rate of gene flow is measured by the introgression probability $\varphi_{XY}$ in MSC-I, which is the proportion of immigrants from the donor population *X* in the recipient population *Y*. In the MSC-M model, the rate of gene flow is measured by the population migration rate $M_{XY}=N_{Y}m_{XY}$, which is the expected number of migrants per generation, where $N_{Y}$ is the population size of the recipient species *Y* and $m_{XY}$ is the proportion of migrants in population *Y*.

For tree B, the species split times were $\tau_{r}=5\theta$, $\tau_{s}=4\theta$, $\tau_{t}=3\theta$, and $\tau_{u}=4.5\theta$. For tree U, they were $\tau_{r}=5\theta$, $\tau_{s}=4\theta$, $\tau_{t}=3\theta$, and $\tau_{u}=2.5\theta$. The introgression times under the MSC-I model were $\tau_{b}=\tau_{c}=\theta$, and $\tau_{d}=\tau_{e}=\theta$. We used two values for *θ*: 0.0025 and 0.01, mimicking data at different mutation rates. In the MSC-I model, we used the introgression probabilities $\varphi_{bc}=0.3$ and $\varphi_{de}=0.2$, while in the MSC-M model, the migration rates were $M_{bc}=m_{bc}N_{C}=0.3$ and $M_{de}=0.2$. Those rates may be representative of gene flow between closely related species, although empirical estimates vary hugely among datasets (e.g. Flouri et al. 2023, Table 1; Thawornwattana et al. 2023b, supplementary fig. S4, Supplementary Material online; Thawornwattana et al. 2023a, supplementary fig. S8, Supplementary Material online; Pavon-Vazquez et al. 2024, table S6, Supplementary Material online).

For each parameter setting, we generated 100 replicate datasets. Each dataset consisted of L=40 or 160 loci, with S=1 or 4 diploid sequences per species at each locus, with the sequence length to be either N=250 or 1,000 sites. In total 3,200 datasets were generated.

Each replicate dataset was analyzed using Bpp v.4.7 (Flouri et al. 2018) to estimate the 21 parameters in the MSC-I model or 15 parameters in the MSC-M model. The correct species tree, gene flow events and mutation model (JC) were assumed. Thus, gene flow rates that were not simulated were not parameters specified in the model and not inferred. For analysis under the MSC-I model, we used the option thetamodel=linked-msci to assign the same population size parameter to the species before and after the introgression event (e.g. branches *tb* and *bB* in Fig. 7a were considered one species). We also applied the Bayesian test of gene flow, calculating the Bayes factor ($B_{10}$) in support of the alternative hypothesis of gene flow ($H_{1}$) over the null hypothesis of no gene flow ($H_{0}$) via the Savage–Dickey density ratio (Ji et al. 2023). This requires the processing of the MCMC sample generated under $H_{1}$, to calculate the posterior probability that the rate of gene flow is very low (see Ji et al. 2023, for details). We used the cut-offs φ<0.005 for the MSC-I model and M<0.005 for the MSC-M model.

As in species tree estimation described above, we also applied two approaches of treating heterozygote sites in the Bpp analysis, either integrating over phase resolutions in the diploid sequence or treating the heterozygote sites in the diploid sequence as ambiguities in a haploid sequence. The two approaches are referred to as Bpp and Bpp-ambiguity, respectively.

We assigned gamma priors on the population size parameters (*θ*) and on the root age for the species tree ($\tau_{0}=\tau_{r}$), with the shape parameter 2 and the prior means equal to the true values: $\tau_{0}∼$ G(2,160) and θ∼ G(2,800) for data simulated at the lower mutation rate (θ=0.0025), and $\tau_{0}∼$ G(2,40) and θ∼ G(2,200) for θ=0.01. The introgression probabilities under MSC-I were assigned the uniform prior beta(1,1), while the migration rates under MSC-M are assumed the prior M∼ G(1,10). While the same *θ* was used for all species on the species tree in the simulation, every branch on the species tree had its own *θ* when the data were analyzed using Bpp.

We used 32,000 iterations for burnin, after which we took $10^{5}$ samples, sampling every two iterations. Analysis of each dataset took ≈4 h on a single thread for small datasets of 40 loci and 10 sequences per locus or ≈23 h for large datasets of 160 loci and 40 sequences per locus.

## Supplementary Material

msaf184_Supplementary_Data

## Data Availability

The Bpp control files and scripts used for simulating and analyzing data in this article are available on Zenodo at https://doi.org/10.5281/zenodo.15790168.
