# Supplementary material for: The Impact of Sequencing and Genotyping Errors on Bayesian Analysis of Genomic Data under the Multispecies Coalescent Model
Source: Mol Biol Evol. 2025 Aug 18;42(8):msaf184. doi: 10.1093/molbev/msaf184 (PMC12359030; doi:10.1093/molbev/msaf184)
Supplement: msaf184_Supplementary_Data [file msaf184_supplementary_data.zip › Ji-2025-bpp-readdepth-SI.pdf]

# Supplemental Information for
## "The impact of sequencing and genotyping errors on Bayesian analysis of genomic data under the multispecies coalescent model"

Jiayi Ji, Paschalia Kapli, Tomáš Flouri, and Ziheng Yang

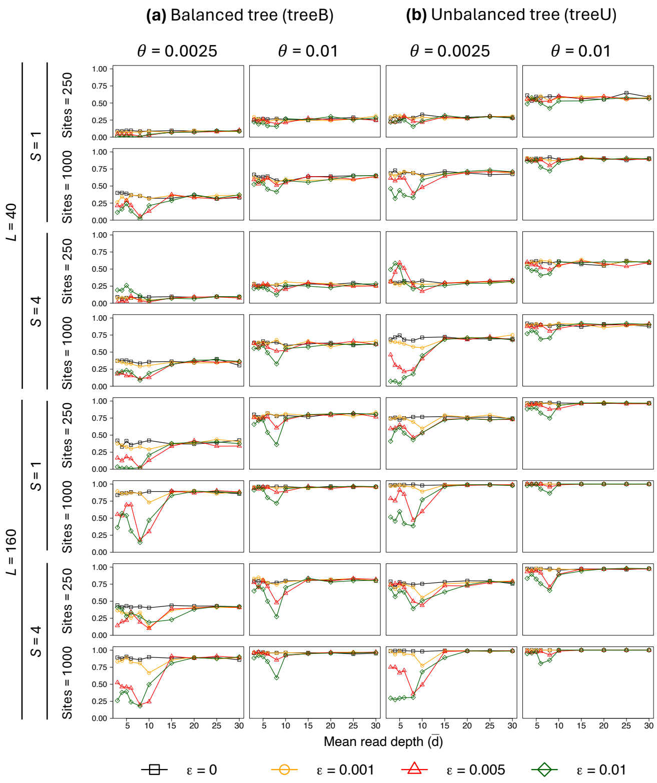


**Fig. S1**: Average posterior probabilities for the correct species tree in BPP species tree estimation using simulated data at different mean read depths ($\bar{d}$) and base-calling error rates ($\epsilon$). The true species trees are trees B and U of figure 3.

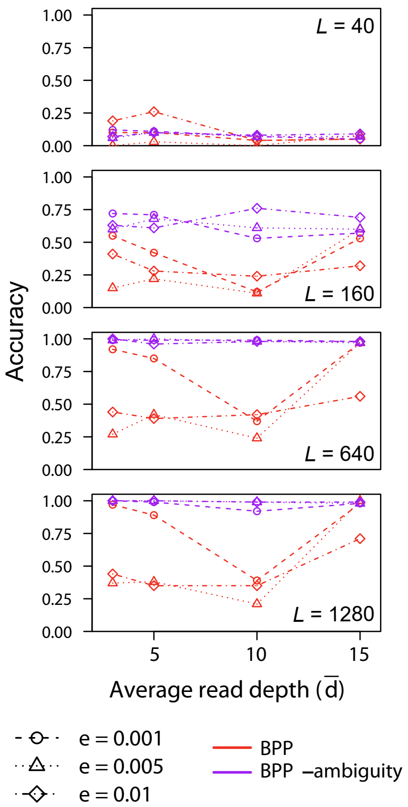


**Fig. S2**: Accuracy of BPP estimation of tree B with $\theta = 0.0025$ at different average read depths ($\bar{d}$) and base-calling error rates ($\epsilon$). Each dataset contains $L = 40, 160, 640$ or $1280$ loci, with $S = 4$ diploid sequences per species per locus and $N = 250$ sites per sequence, simulated using the average read depth $\bar{d} = 3, 5, 10, 15$ and base-calling error rate $\epsilon = 0.001, 0.005, 0.01$.

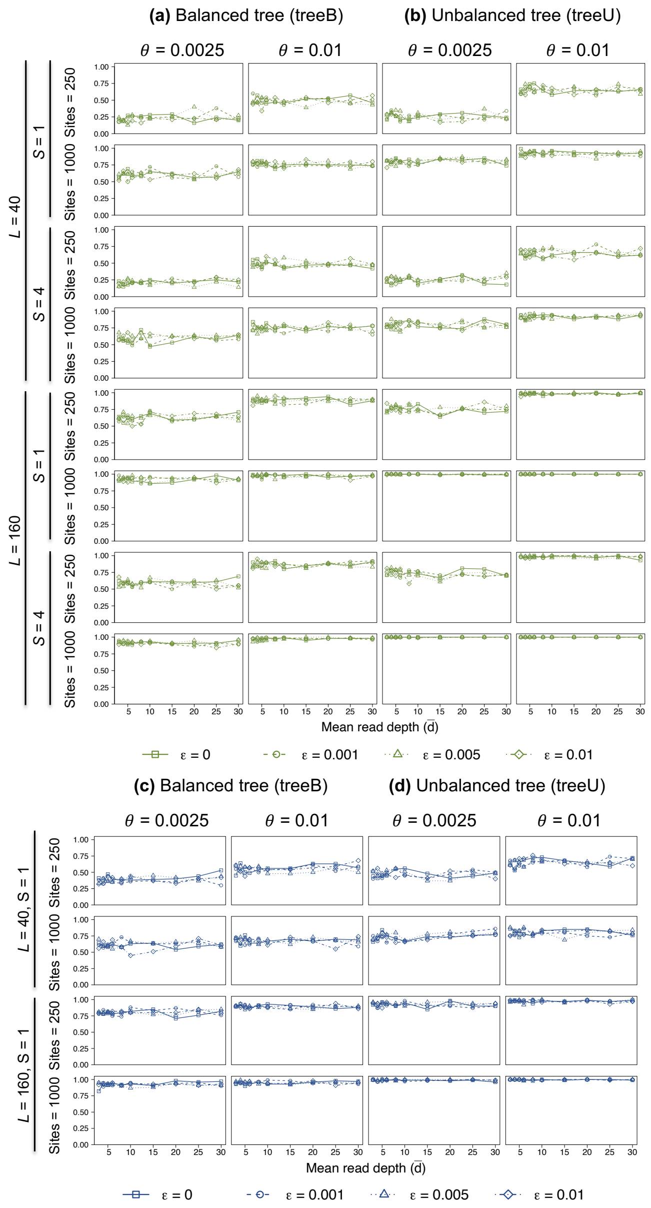


**Fig. S3**: Accuracy of species tree estimation using (**a**, **b**) ASTRAL and (**c**, **d**) concatenation/ML at different mean read depths ($\bar{d}$) and base-calling error rates ($\epsilon$). Data are the same as those of figure 4 except that an outgroup ($O$) is also included to root the tree. Concatenation/ML is applied to data of one (diploid) sequence per species ($S = 1$) only.

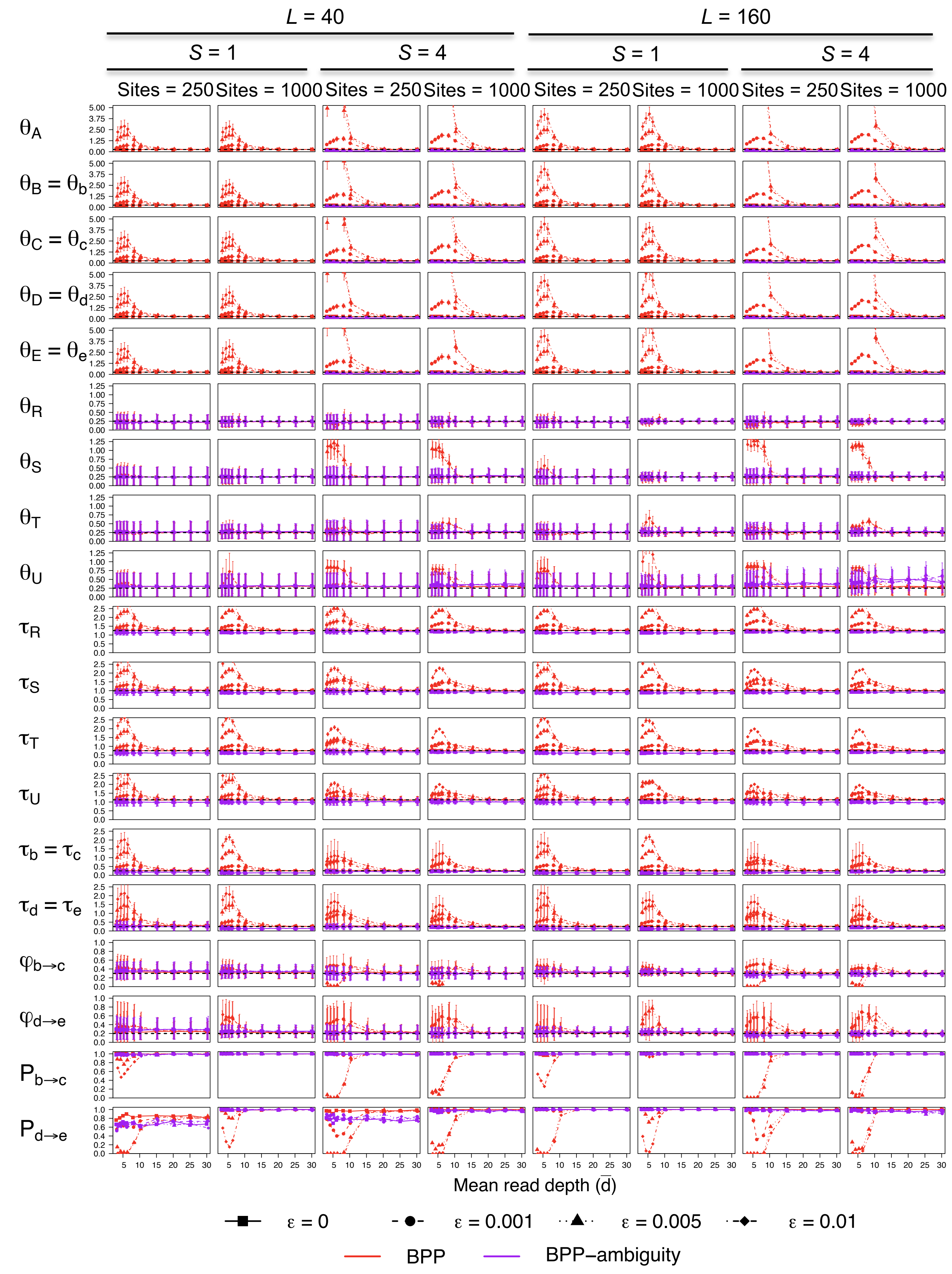


**Fig. S4**: [I-B-0.0025] Average posterior means and 95% HPDs for parameters under the MSC-I model of tree B (fig 7a) with $\theta$ = 0.0025. See legend to figure 8.

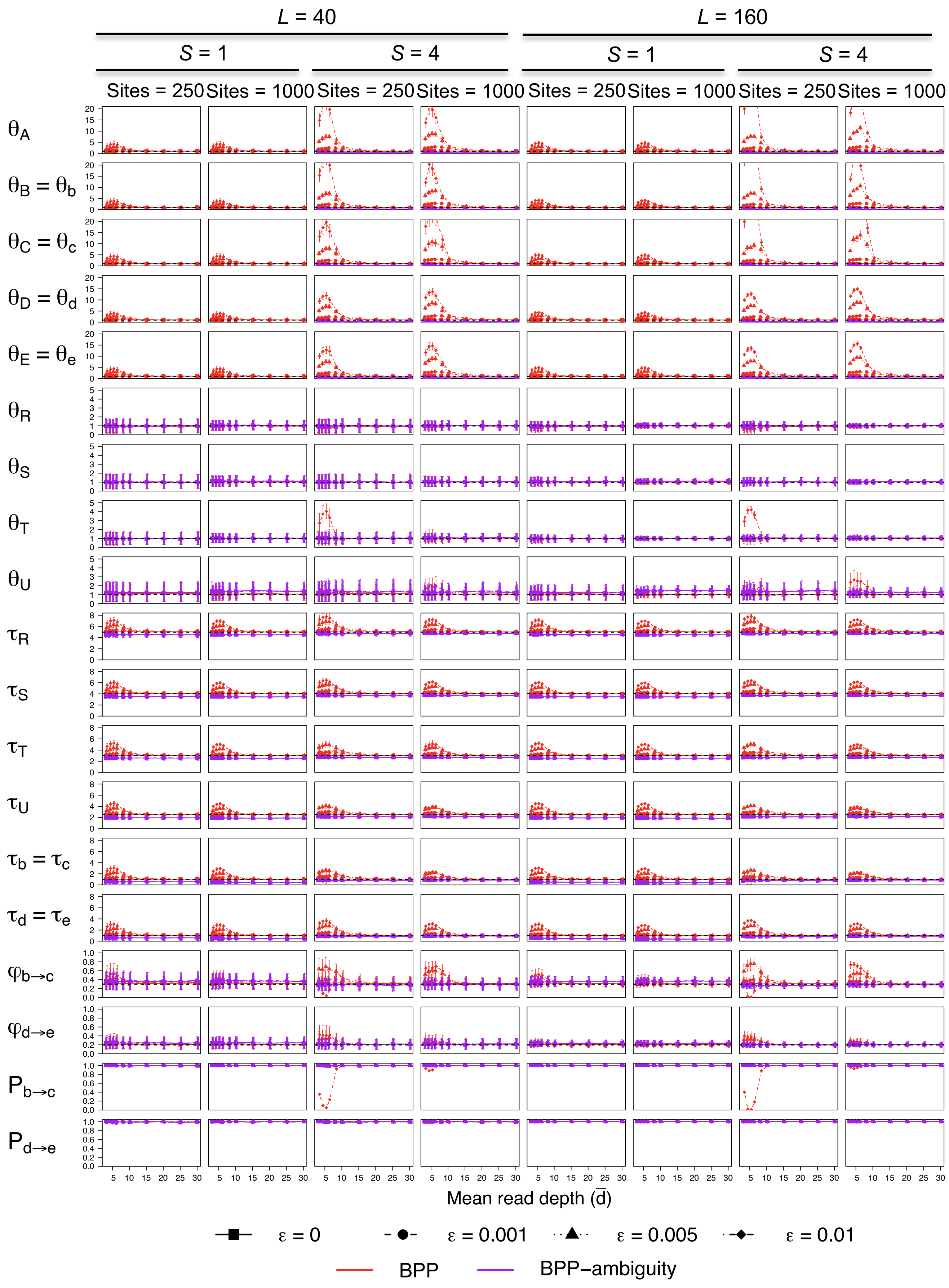


**Fig. S5**: [I-U-0.01] Average posterior means and 95% HPDs for parameters under the MSC-I model of tree U (fig. 7b) with $\theta = 0.01$. See caption to figure 8.

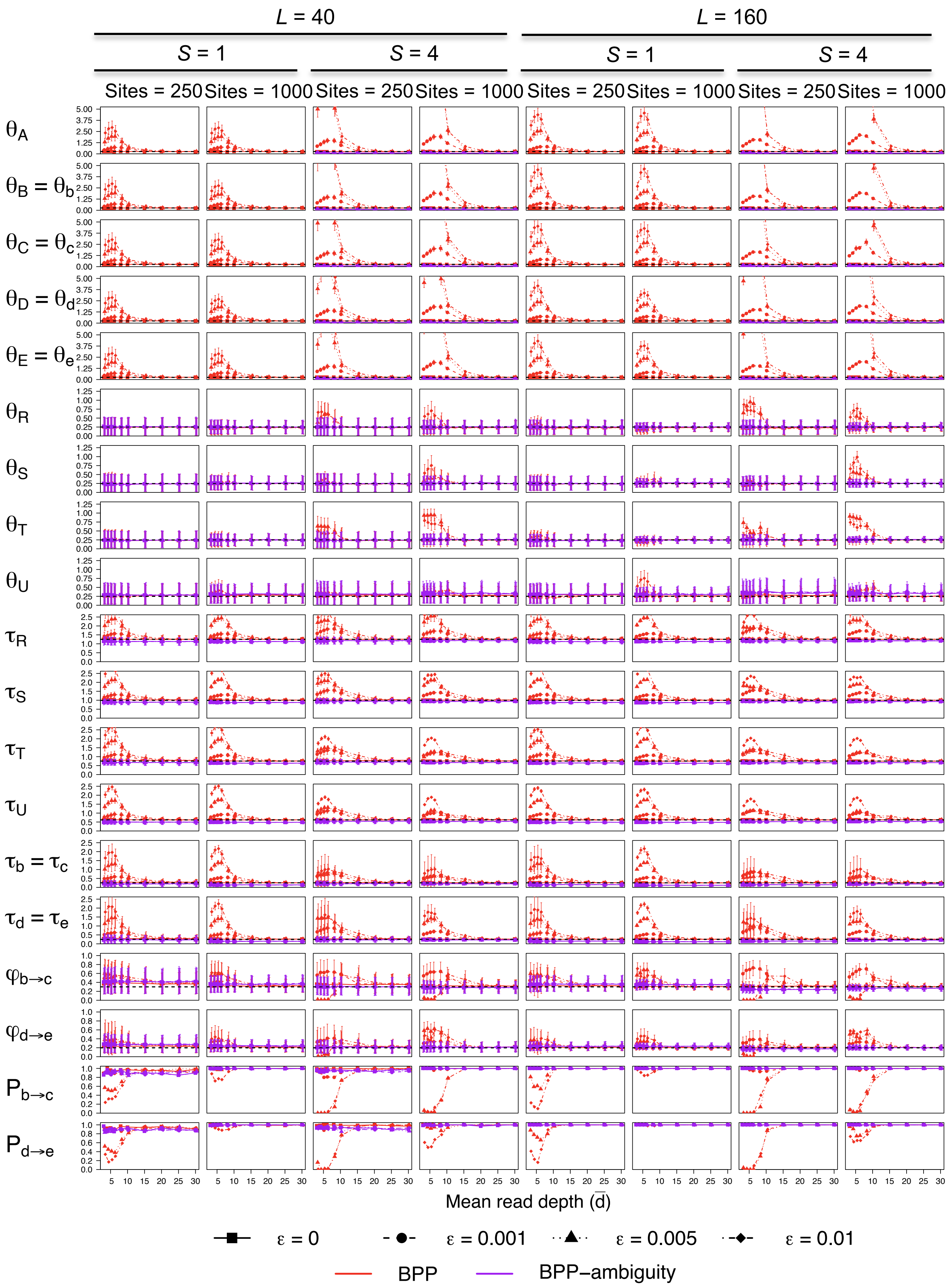


**Fig. S6**: [I-U-0.0025] Average posterior means and 95% HPDs for parameters under the MSC-I model of tree U (fig. 7b) with $\theta = 0.0025$. See caption to figure 8.

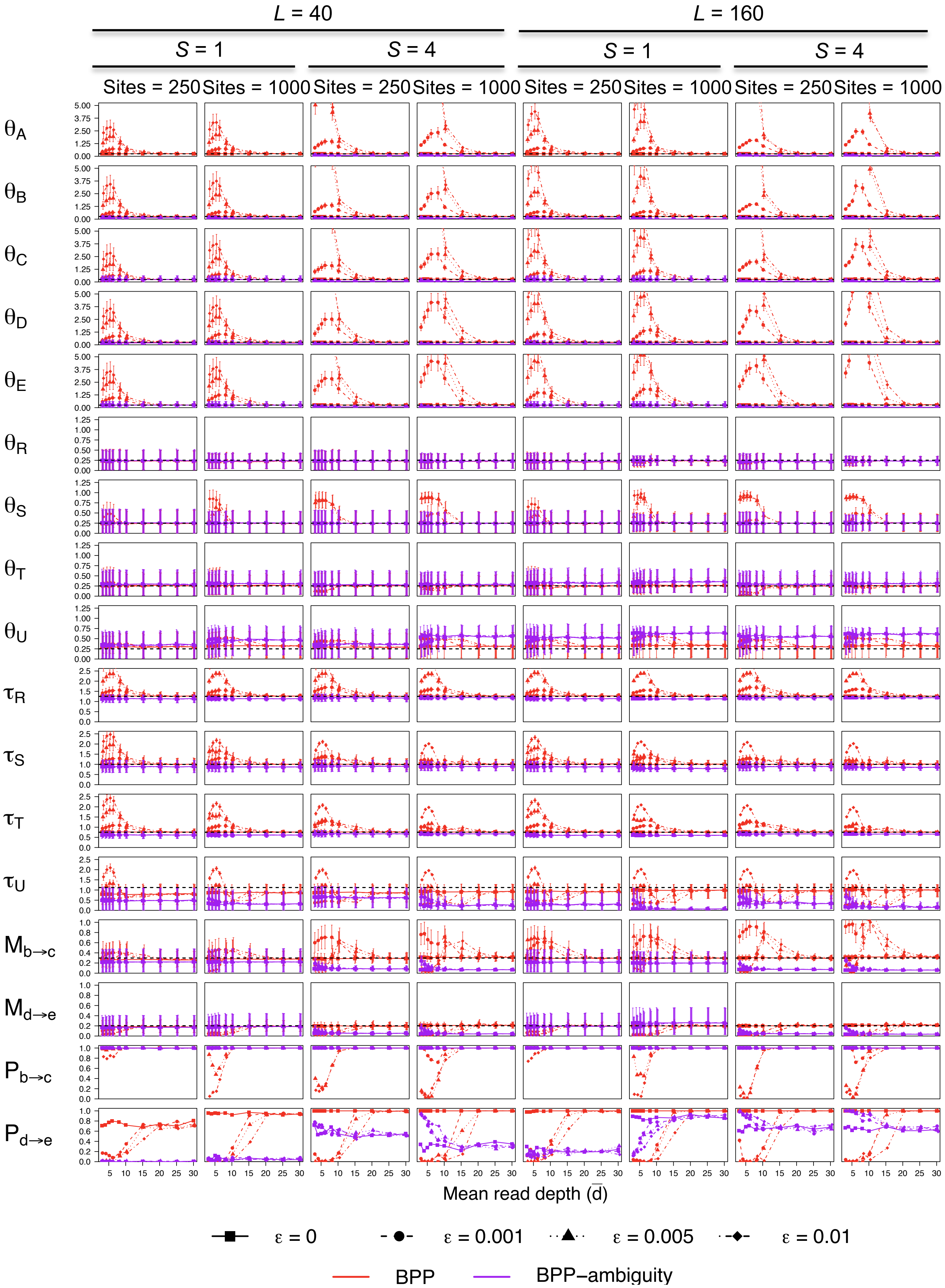


**Fig. S7**: [M-B-0.0025] Average posterior means and 95% HPDs for parameters under the MSC-M model of tree B (fig. 7a) with $\theta$ = 0.0025. See caption to figure 8.

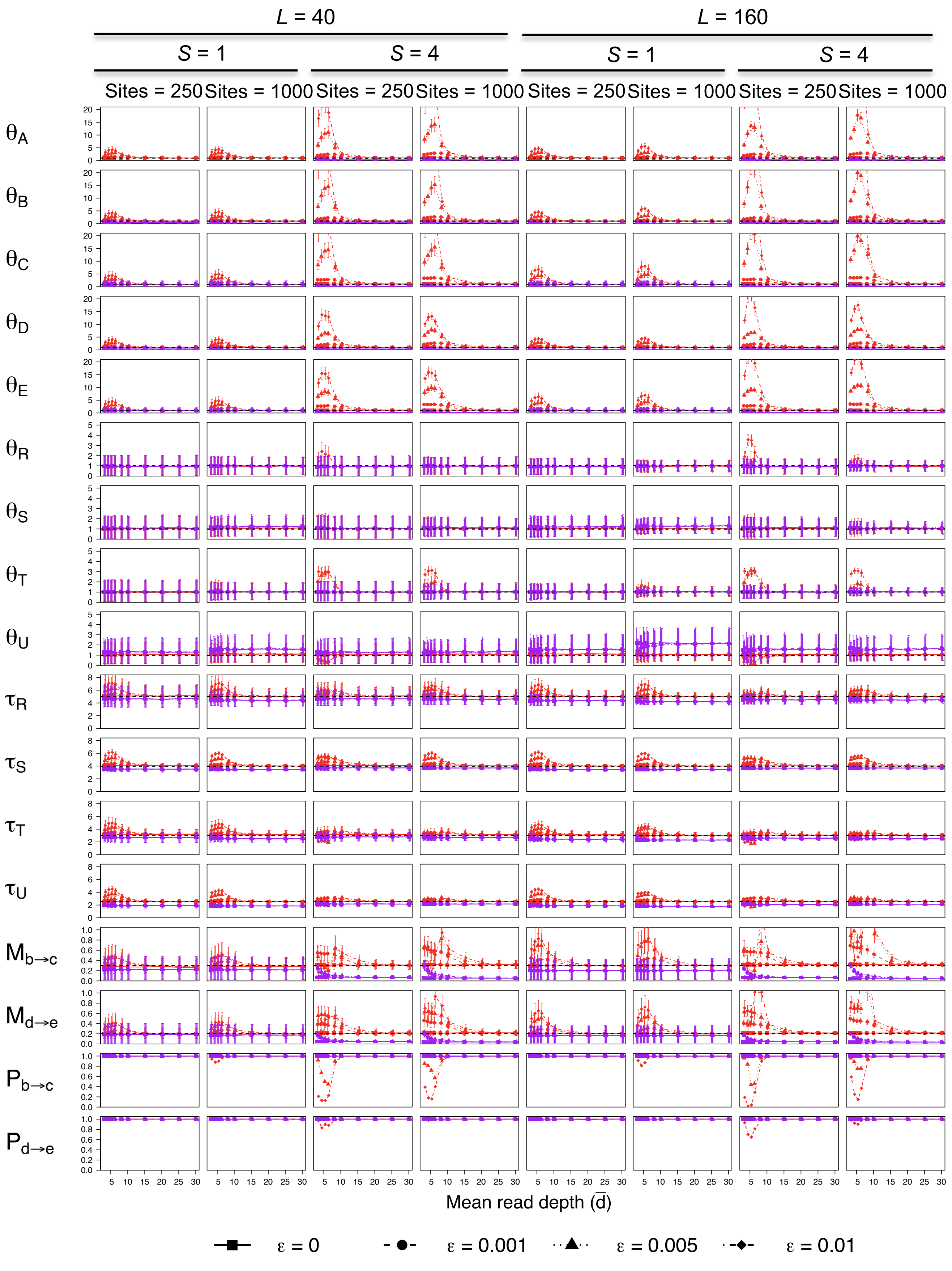


**Fig. S8**: [M-U-0.01] Average posterior means and 95% HPDs for parameters under the MSC-M model of tree U (fig. 7b) with $\theta = 0.01$. See caption to figure 8.

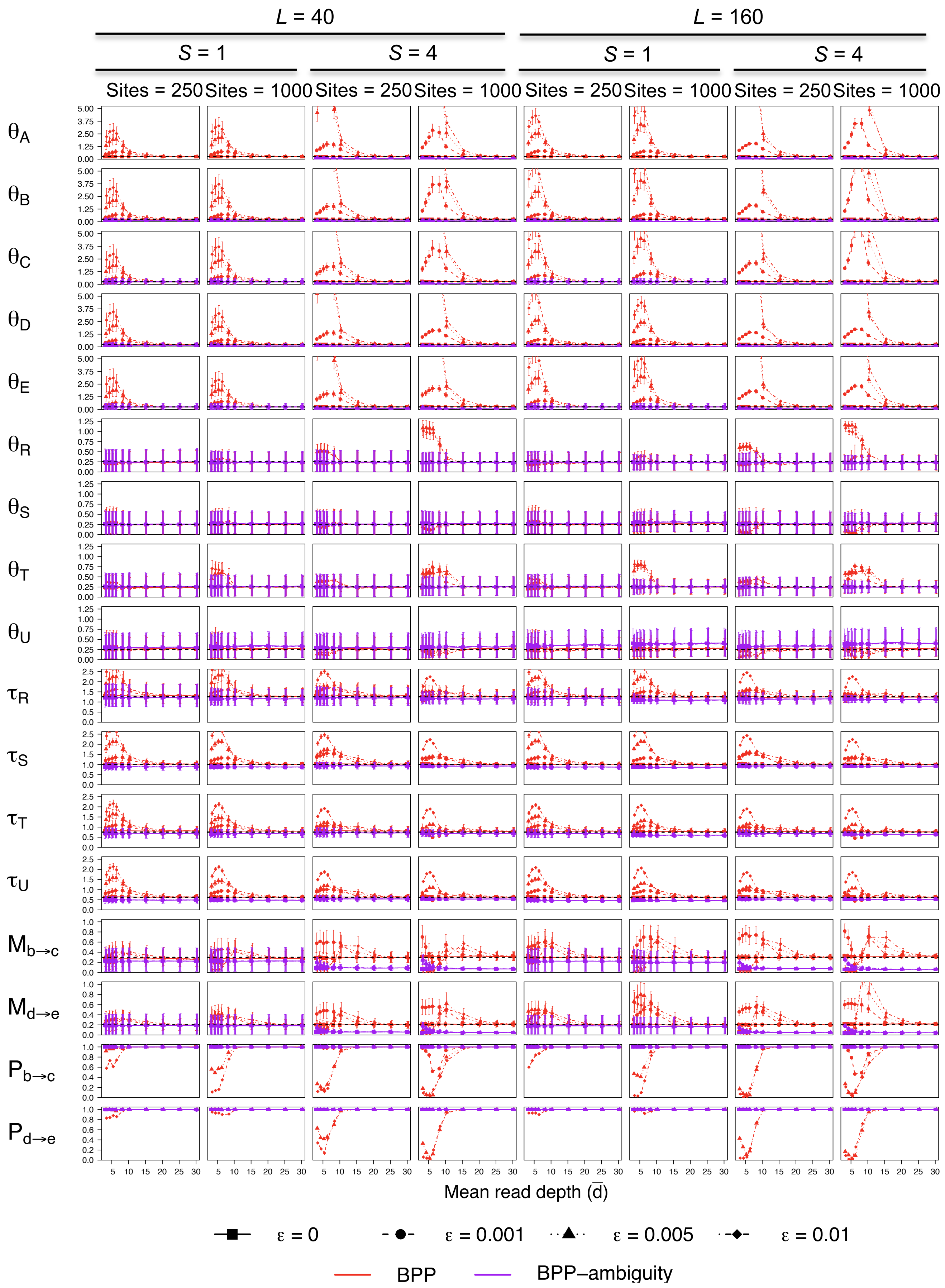


**Fig. S9**: [M-U-0.0025] Average posterior means and 95% HPDs for parameters under the MSC-M model of tree U (fig. 7b) with $\theta$ = 0.0025. See caption to figure 8.

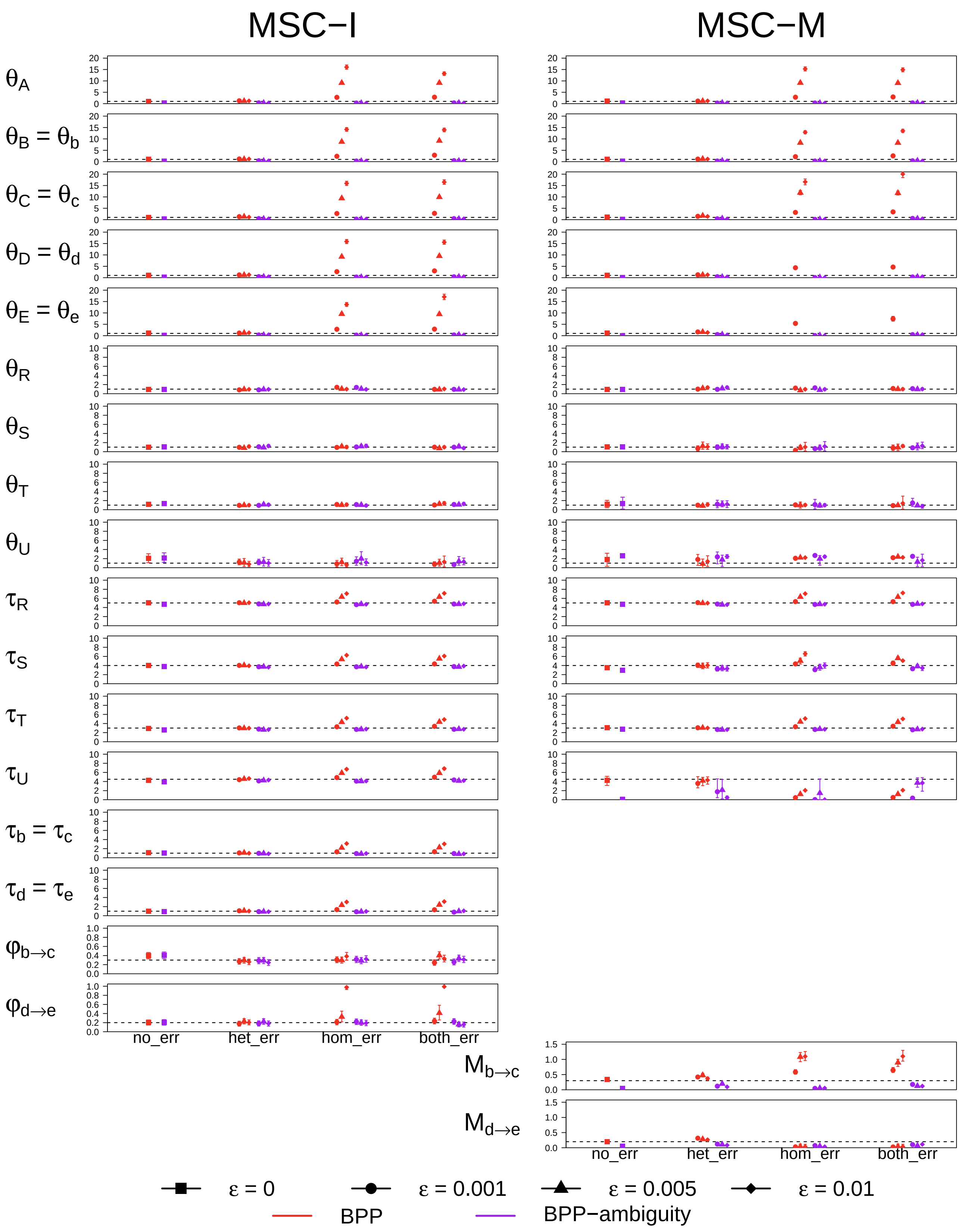


**Fig. S10**: Posterior means and 95% HPD CIs for parameters in the MSC-I and MSC-M models (fig. 7**a**&**b**) in BPP analysis of simulated datasets with no, one, or both types of genotyping errors: homozygotes miscalled as heterozygotes (hom-err) and heterozygotes miscalled as homozygotes (het-err). Each dataset consists of $L = 160$ loci, with $S = 4$ diploid sequences per species per locus, and $N = 1000$ sites per sequence, simulated using the average read depth $\bar{d} = 5$, and the balanced tree B at the high mutation rate ($\theta = 0.01$). Results for the settings of no errors (no-err) and both types of errors (both-err) are from figures 8 (I-B-0.01) and 9 (M-B-0.01).
